# Review of the southern African slender stonebashers, genus *Heteromormyrus* Steindachner 1866 (Teleostei: Mormyridae), with description of six new species

**DOI:** 10.1111/jfb.70191

**Published:** 2025-09-17

**Authors:** Tadiwa I. Mutizwa, Wilbert T. Kadye, Pedro H. N. Bragança, Albert Chakona

**Affiliations:** ^1^ Department of Ichthyology and Fisheries Science Rhodes University Makhanda South Africa; ^2^ National Research Foundation – South African Institute for Aquatic Biodiversity (NRF‐SAIAB) Makhanda South Africa; ^3^ Department of Ichthyology American Museum of Natural History New York New York USA

**Keywords:** diversity, freshwater, mormyrids, southern Africa, taxonomy

## Abstract

Recent molecular studies have advanced our knowledge of the taxonomic diversity and generic placement of the slender stonebashers, previously placed in the genus *Hippopotamyrus*, in southern Africa. These fishes were recently transferred to the genus *Heteromormyrus* whose range encompasses the Kwanza, Kunene, Okavango, Zambezi, Pungwe and Buzi River systems in southern Africa, as well as the southern tributaries of the Congo River system. The present study builds on previous research that identified at least eight candidate species within the *Heteromormyrus ansorgii* species complex by providing formal descriptions for six new species and redescriptions of *Heteromormyrus pauciradiatus* and *H. ansorgii* s.s. The Kwanza River system is peculiar because it currently has five known species in this genus, some of which are co‐distributed, whereas the other river systems have only one or two species in this genus. Two of the new species, *Heteromormyrus dolichorhynchus* sp. nov. and *Heteromormyrus angusticaudata* sp. nov., are endemic to the Kwanza River system. *Heteromormyrus xanekweorum* sp. nov. is confined to the Okavango River system, *Heteromormyrus chilembwei* sp. nov. occurs in the Ruo River (lower Zambezi River system), *Heteromormyrus tangwenai* sp. nov. is endemic to the Pungwe River system and *Heteromormyrus ndauorum* sp. nov. is endemic to the Buzi River system. Species in this genus exhibit high morphological similarity, but they can be separated by a combination of characters, including scale counts, dorsal‐ and anal‐fin ray counts, vertebral counts, caudal peduncle depth, position of nostrils, head shape and variation in colour pattern. Taxonomic diversity within this genus is likely to be higher than currently known, and future studies, particularly in the Kwanza and upper Zambezi rivers, are anticipated to uncover additional new species.

## 1 INTRODUCTION

The Mormyridae Bonaparte 1831 is the most diverse family within the order Osteoglossiformes, comprising 227 valid species in 22 genera (Fricke et al., 2024; Nelson, 2016). Fishes of this family are endemic to Africa where they are found in several rivers across the continent, with the highest diversity occurring in Central and West Africa (Peterson et al., 2022; Roberts, 1975; Skelton, 2001). These fishes are known for their ability to produce and detect weak electric organ discharges (EODs), a key evolutionary innovation that they use for electrolocation and electrocommunication (Arnegard et al., 2010; Carlson & Arnegard, 2011; Feulner et al., 2007; Kramer, 1990; Lavoué et al., 2003; Lissmann, 1958; Sullivan et al., 2000). Evidence from several mormyrid species has shown that EODs diverged more rapidly than other ecomorphological characters, thereby potentially forming early reproductive barriers between species (Arnegard et al., 2010; Arnegard & Hopkins, 2003; Carlson & Arnegard, 2011; Feulner et al., 2008, 2009). This makes the delimitation of species boundaries among some closely related mormyrid species using only traditional morphological approaches challenging. Consequently, the species‐level taxonomy in Mormyridae remains poorly resolved, as shown by the presence of taxonomic conflicts in most genera of this family (Peterson et al., 2022). For example, in southern Africa, the Mormyridae are represented by eight genera with a total of 27 species, most of which are thought to be broadly distributed across multiple river systems (Marshall, 2011; Skelton, 2001). Recent studies implementing integrated taxonomic approaches have identified several undescribed species that were previously included within a single widely distributed species, highlighting the need for further reviews of the taxonomy of the mormyrid fishes within this region (Kramer et al., 2003, 2007, 2012, 2016; Kramer & Swartz, 2010; Kramer & van der Bank, 2000; Kramer & van der Bank, 2011; Kramer & Wink, 2013; Maake et al., 2014).

Advances in molecular approaches have facilitated the resolution of mormyrid taxonomy. For example, five species of slender stonebashers from southern Africa, including *Heteromormyrus ansorgii* (Boulenger, 1905) and *Heteromormyrus pappenheimi* (Boulenger, 1910), both occurring in the Kwanza River system; *Heteromormyrus tavernei* (Poll, 1972) from the upper Lualaba and Lufira rivers; *Heteromormyrus longilateralis* (Kramer & Swartz, 2010), a species endemic to the Kunene River system; and *Heteromormyrus szaboi* (Kramer et al., 2004) found throughout the upper Zambezi River system, which were previously included in the genera *Hippopotamyrus* Pappenheim, 1906 and *Brienomyrus* Taverne, 1971, were transferred into the genus *Heteromormyrus* Steindachner, 1866. This transfer of species was based on mitogenomic data demonstrating the monophyly of the slender stonebashers from southern African (all but *H. pappenheimi*, which was not sequenced) with the holotype of *Heteromormyrus pauciradiatus* Steindachner, 1866, while showing their polyphyletic relationship with the type species of *Hippopotamyrus* from Cameroon (Sullivan et al., 2022).

Several previous studies on *H. ansorgii* indicated that it is a species complex concealing undocumented diversity across its disjunct distribution range (Chakona et al., 2018; Kramer et al., 2004; Kramer & Swartz, 2010; van der Bank & Kramer, 1996). This species, originally described based on two specimens collected from an uncertain locality broadly identified as ‘between Benguella and Bihé’, in Angola, was subsequently reported from several southern African river systems, including the Kwanza, Kunene, Okavango, Zambezi, Pungwe and Buzi rivers (Bell‐Cross & Minshull, 1988; Chakona et al., 2018; Kadye et al., 2008; Marshall, 2011; Skelton, 2001). Mutizwa et al. (2021), through detailed analysis of comprehensive molecular and morphological data, identified 10 distinct lineages within the *H. ansorgii* complex, and resolved that the type locality of this species was likely to be in the Kwanza River system. Five of the lineages identified by Mutizwa et al. (2021) as K1*–*K5 are distributed in the Kwanza River system. Subsequently, Sullivan et al. (2022) provided mitogenomic evidence that indicated that one of the Kwanza River lineages (K4) represents *H. pauciradiatus*. However, it remains unclear which of the other lineages identified from the Kwanza River represent the two species that were described from this system, *H. ansorgii* and *H*. *pappenheimi*.

The purpose of this study was to undertake detailed morphological examination of the lineages within the *H. ansorgii* complex identified by Mutizwa et al. (2021) to identify diagnostic characters that support their recognition and description as new species. In the Kwanza River system, there are five lineages within the *H. ansorgii* complex (K1–K5), and three names are available (*H. pauciradiatus*, *H. ansorgii* and *H. pappenheimi*). Sullivan et al. (2022) provided evidence showing that the K4 lineage represents *H. pauciradiatus*. For the present study, our first objective was to determine if any of the identified lineages from this system are conspecific with either *H. ansorgii* or *H. pappenheimi*. The second objective was to provide redescriptions of *H. ansorgii s.s*. and *H. pauciradiatus*, and describe six new species: two from the Kwanza River system, and one species each from the Okavango, Ruo, Pungwe and Buzi river systems. Two lineages included in Mutizwa et al. (2021), *Heteromormyrus* sp. ‘K2’ from the Kwanza River and *Heteromormyrus* sp. ‘UZ1’ from the upper Zambezi River, were not included in the present study because they were only represented by juvenile specimens. This study builds on the previous work that highlighted the hidden diversity in the genus *Heteromormyrus* (Chakona et al., 2018; Kramer et al., 2004; Kramer & Swartz, 2010; Mutizwa et al., 2021) by providing formal names for most of the known lineages in southern Africa, thus ensuring their inclusion in future studies and conservation planning for this region.

## 2 MATERIALS AND METHODS

2.1

This study included specimens obtained from the National Research Foundation – South African Institute for Aquatic Biodiversity (NRF‐SAIAB), in Makhanda, South Africa. Ethical clearance for this research was granted by the Rhodes University Animal Ethics Committee (reference number: 2700). The sampling approaches were approved by the NRF‐SAIAB Animal Ethics Committee (reference number: 2014/03). Permission to survey localities in the Eastern Highlands of Zimbabwe was approved by the Zimbabwe Parks and Wildlife Authority.

### 2.2 Examined material

The present study was based on specimens collected from across the range of the *H. ansorgii* species complex during surveys conducted between 1999 and 2017. Most specimens were collected using a combination of seine netting and electrofishing. Specimens were euthanized with clove oil, and a small piece of muscle tissue or fin clip for genetic analysis was dissected from some specimens and placed in a tube with 99% ethanol and stored at −80°C for long‐term storage. Voucher specimens were fixed in formaldehyde before being stored in 70% ethanol for long‐term storage. Voucher specimens and tissue samples were deposited into the National Fish Collection and the National Biobank, both at NRF‐SAIAB, Makhanda, South Africa.

This study examined 174 specimens from 50 collection sites. These specimens were obtained from NRF‐SAIAB, the Royal Museum for Central Africa in Tervuren, Belgium, (RMCA) and the Natural History Museum (BMNH) in London, UK. The specimens represented different *H. ansorgii* lineages identified by Mutizwa et al. (2021): *Heteromormyrus* sp. ‘Buzi’ (*n* = 7) from the Buzi River system, *Heteromormyrus* sp. ‘Pungwe’ (*n* = 18) from the Pungwe River system, *Heteromormyrus* sp. ‘Ruo’ (*n* = 7) from the Rou River, lower Zambezi system, *Heteromormyrus* sp. ‘OK’ (*n* = 36) from the Okavango River system, *Heteromormyrus* sp. ‘K1’ (*n* = 8), *Heteromormyrus* sp. ‘K3’ (*n* = 16) and *Heteromormyrus* sp. ‘K5’ (*n* = 23) from the Kwanza River system. Comparative material included *H. ansorgii* syntypes (*n* = 2), *H. szaboi* (*n* = 13), *H. longilateralis* (*n* = 11), *H. pauciradiatus* (*n* = 25) and *H. pappenheimi* syntypes (*n* = 8). In this study, we examined and measured the two syntypes of *H. ansorgii*. However, we could not access the syntypes of *H. pappenheimi*. We therefore made comparisons based on meristic data from X‐ray radiographs and the original description by Boulenger (1910). Similarly, data for *H. tavernei* were obtained from the original description by Poll (1972). Detailed information of the specimens examined is presented in the material examined section of each species account.

### 2.3 Morphological analyses

**FIGURE 1:**
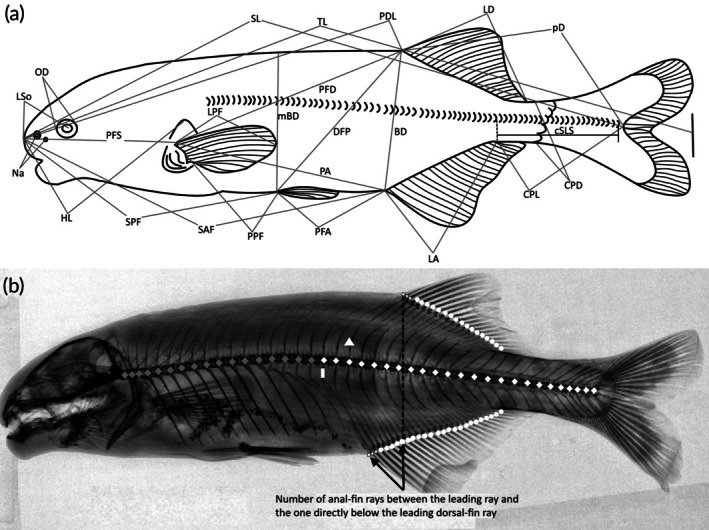
(a) Illustration of the morphological characters examined in this study. Explanation for the morphometric characters provided in the materials and methods section. (b) Radiograph showing how osteological counts were done in the present study: 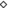
pre‐caudal vertebrae (PC), 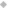
caudal vertebrae (CV), 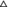
vertebra before the first dorsal‐fin radial (FDR), 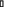
vertebra before the first anal‐fin radial (FAR), 
unbranched fin rays, 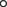
branched fin rays and first anal‐fin ray to first dorsal‐fin ray (AD).

Measurements and external counts were done on the left side of each specimen. Digital Vernier callipers were used for measurements to the nearest 0.1 mm following Mutizwa et al. (2021), with the addition of six osteological characters obtained from X‐ray radiographs and the exclusion of three morphometric characters (distance from upper lip to centre of orbit, distance from mouth edge to centre of orbit, distance from mental lobe to centre of orbit) that were difficult to constantly measure. The study examined 26 point‐to‐point measurements and 15 meristic counts (Figure 1a,b). These measurements and meristic counts included total length (TL), standard length (SL), pre‐dorsal length (PDL), pre‐anal length (SAF), pre‐pelvic length (SPF), pre‐pectoral length (PFS), pectoral fin to anal fin (PA), pelvic fin to anal fin (PFA), pectoral fin to dorsal fin (PFD), dorsal fin to pelvic fin (DFP), dorsal‐fin base length (LD), anal‐fin base length (LA), dorsal fin to caudal fin (pD), caudal peduncle length (CPL), caudal peduncle depth (CPD), pectoral‐fin length (LPF), pectoral fin to pelvic fin (PPF), body depth (BD), mid body depth (mBD), head length (HL), length of snout (LSo), inter‐nostril distance on the same side (Na), orbit diameter (OD), head width (HW), distance between anterior nostrils (IN), interorbital width (IOW), dorsal‐fin rays (nD), anal‐fin rays (nA), number of anal‐fin rays between the leading ray and the one directly below the leading dorsal‐fin ray (AD), pelvic‐fin rays (nP), pectoral‐fin rays (PFc), number of scales around the caudal peduncle (SPc) and number of scales along the caudal peduncle (cSLS), the lateral‐line scales (SLS), upper‐jaw teeth (UJ), lower‐jaw teeth (LJ) and total vertebrae (TV), pre‐caudal vertebrae (PC), caudal vertebrae (CV), number of vertebrae before the first dorsal‐fin radial (FDR) and number of vertebrae before the first anal‐fin radial (FAR). Vertebrae, dorsal‐ and anal fin‐rays were counted from radiographs taken using the Inspex 20i Digital X‐ray Imaging System (Kodex Inc., New Jersey, NJ, USA) at NRF‐SAIAB, RMCA and BMNH. Vertebrae counts did not include the demi‐centrum fused to the hypural plate. In mormyrids, the first two dorsal‐ and anal‐fin rays are unbranched, unsegmented and so small that they are difficult to see without a radiograph. These two unbranched and unsegmented rays are followed by a single unbranched, segmented ray (the first long ray) followed by numerous branched, segmented rays. The last dorsal‐ and anal‐fin ray is usually branched all the way to its base. In the present study, all rays, both unbranched and branched, were included in the counts. The last ray, which is branched to its base, was counted as one.

## 3 RESULTS

### 3.1 Meristic data of the southern African species of *Heteromormyrus*

**FIGURE 2:**
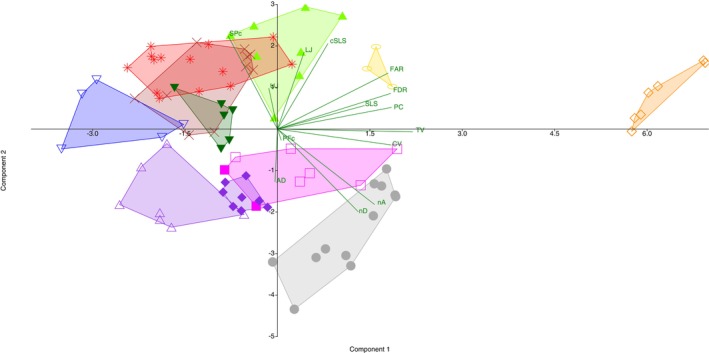
Principal component analysis of the meristic data of the *Heteromormyrus* lineages from southern Africa showing components 1 and 2: pectoral‐fin rays (PFc), number of scales around the caudal peduncle (SPc), number of scales along the caudal peduncle (cSLS), lateral‐line scales (SLS), first anal‐fin ray to first dorsal‐fin ray (AD), pre‐caudal vertebrae (PC), caudal vertebrae (CV), number of vertebrae before the first dorsal‐fin radial (FDR), number of vertebrae before the first anal‐fin radial (FAR), total vertebrae (TV), dorsal‐fin rays (nD), anal‐fin rays (nA), lower jaw teeth (LJ) and upper jaw teeth (UJ). *Heteromormyrus ansorgii* syntype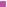, *Heteromormyrus szaboi* 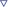, *Heteromormyrus longilateralis* 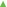, *Heteromormyrus pauciradiatu* 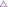, *H. pappenheimi* syntype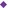, *Heteromormyrus* sp. ‘Buzi’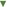, *Heteromormyrus* sp. ‘Pungwe’, *Heteromormyrus* sp. ‘OK’, *Heteromormyrus* sp. ‘Ruo’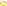, *Heteromormyrus* sp. ‘K1’ 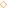, *Heteromormyrus* sp. ‘K3’, *Heteromormyrus* sp. ‘K5’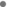.

**FIGURE 3:**
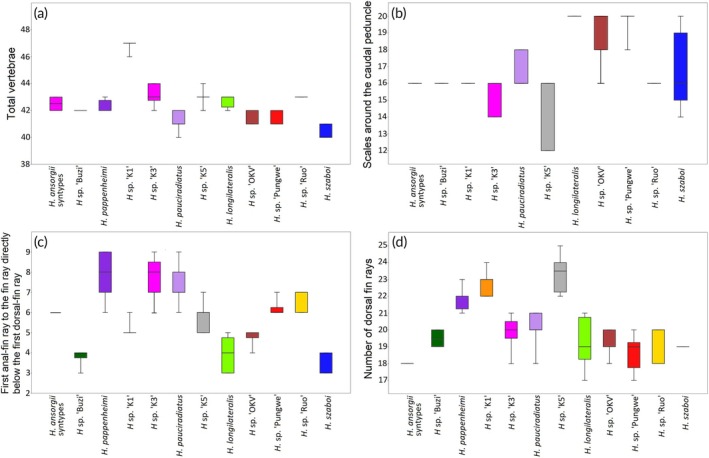
Boxplots of meristic characters that distinguished some of the *Heteromormyrus* lineages and species from southern Africa; the colours are consistent for each lineage or species.

Principal component analysis of the meristic data showed separation between most of the *Heteromormyrus* species. The *H. ansorgii* and *H. pappenheimi* syntypes overlapped, and they were negatively associated with the first principal component (PCI), separating them from *H. szaboi*, which was more negatively associated with this axis, as well as *Heteromormyrus* sp. ‘K1’ and *Heteromormyrus* sp. ‘Ruo’ that were positively associated with this axis (Figure 2). The PCI accounted for 35.7% of the observed variance, and it was positively associated with the total number of vertebrae (Table 1). Consistent with the pattern shown by PCI, *H. ansorgii* (42–43) and *H. pappenheimi* (42–43) have more vertebrae than *H. szaboi* (40–41) but have fewer than *Heteromormyrus* sp. ‘K1’ (46–47) (Figure 3a).

**TABLE 1:** Principal component loadings of the first and second axes of meristic data from *Heteromormyrus* species and lineages from southern Africa.

Principal component	1	2
Eigenvalue	5	2.79
% variance	35.71	19.95
Pectoral‐fin rays (PFc)	0.01	−0.01
Number of scales around the caudal peduncle (SPc)	−0.15	0.45
Number of scales along the caudal peduncle (cSLS)	0.17	0.4
Lateral‐line scales (SLS)	0.28	0.1
First anal‐fin ray to first dorsal‐fin ray (AD)	−0.01	−0.24
Pre‐caudal vertebrae (PC)	0.36	0.1
Caudal vertebrae (CV)	0.36	−0.08
Number of vertebrae before the first dorsal‐fin radial (FDR)	0.36	0.17
Number of vertebrae before the first anal‐fin radial (FAR)	0.35	0.26
Total vertebrae (TV)	0.43	−0.01
Dorsal‐fin rays (nD)	0.26	−0.41
Anal‐fin rays (nA)	0.31	−0.35
Lower‐jaw teeth (LJ)	0.09	0.37
Upper‐jaw teeth (UJ)	−0.04	0.17

The *H. ansorgii* and *H. pappenheimi* syntypes overlapped and were negatively associated with the second principal component (PCII), separating them from *H. longilateralis*, *Heteromormyrus* sp. ‘Pungwe’, *Heteromormyrus* sp. ‘K1’, *Heteromormyrus* sp. ‘Ruo’ and *Heteromormyrus* sp. ‘OK’, which were positively associated with this axis (Figure 2). The PCII accounted for 20.0% of the observed variance, and it was positively associated with the number of scales around the caudal peduncle (Table 1). Consistent with the pattern shown by PCII, *H. pappenheimi*, a species with a low number of scales around the caudal peduncle (12–14), is separated from *H. pauciradiatus* (16–18), *H. tavernei* (20–22), *H. longilateralis* (20), *Heteromormyrus* sp. ‘Pungwe’ (18–20), *Heteromormyrus* sp. ‘K1’ (16), *Heteromormyrus* sp. ‘OK’ (16–20), *Heteromormyrus* sp. ‘Ruo’ (16) and *Heteromormyrus* sp. ‘Buzi’ (16, Figure 3b). Similarly, the scales around the caudal peduncle separate *H. ansorgii* (16) from *H. longilateralis* (18–20) and *Heteromormyrus* sp. ‘Pungwe’ (18–20, Figure 3b). Additionally, the number of anal‐fin rays between the leading ray and the one directly below the leading dorsal‐fin ray in the *H. ansorgii* (6) and *H. pappenheimi* (6–9) syntypes distinguish them from *H. longilateralis* (3–5), *H. szaboi* (3–4), *Heteromormyrus* sp. ‘OK’ (4–5) and *Heteromormyrus* sp. ‘Buzi’ (3–4, Figure 3c). The number of dorsal‐fin rays distinguish *H. ansorgii* syntypes (18) from the *H. pappenheimi* (21–23) syntypes, *Heteromormyrus* sp. ‘K1’ (22*–*24) and *Heteromormyrus* sp. ‘K5’ (22*–*25, Figure 3d).

### 3.2 Morphometric data of the southern African species of *Heteromormyrus*

**FIGURE 4:**
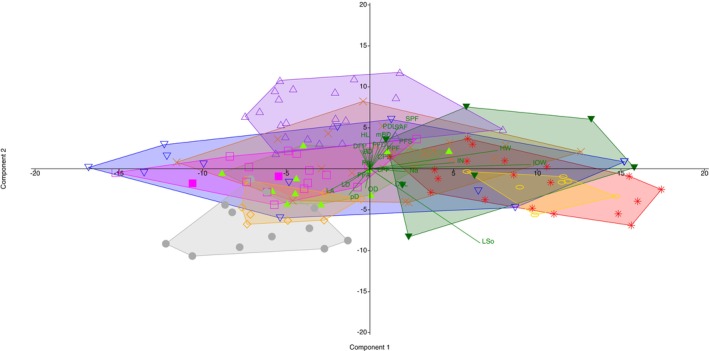
Principal component analysis of the morphometric data of the *Heteromormyrus* lineages from southern Africa showing components 1 and 2: pre‐dorsal length (PDL), pre‐anal length (SAF), pre‐pelvic length (SPF), pre‐pectoral length (PFS), pectoral fin to anal fin (PA), pelvic fin to anal fin (PFA), pectoral fin to dorsal fin (PFD), dorsal fin to pelvic fin (DFP), dorsal‐fin base length (LD), anal‐fin base length (LA), dorsal fin to caudal fin (pD), caudal peduncle length (CPL), caudal peduncle depth (CPD), pectoral‐fin length (LPF), pectoral fin to pelvic fin (PPF), body depth (BD), mid body depth (mBD), head length (HL), length of snout (LSo), distance between nostrils on the same side (Na), orbit diameter (OD), head width (HW), distance between anterior nostrils (IN) and interorbital width (IOW). *Heteromormyrus ansorgii* syntype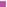, *Heteromormyrus szaboi* 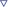, *Heteromormyrus longilateralis* 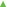, *Heteromormyrus pauciradiatus* 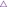, *Heteromormyrus* sp. ‘Buzi’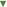, *Heteromormyrus* sp. ‘Pungwe’, *Heteromormyrus* sp. ‘OK’, *Heteromormyrus* sp. ‘Ruo’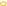, *Heteromormyrus* sp. ‘K1’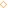, *Heteromormyrus* sp. ‘K3’, *Heteromormyrus* sp. ‘K5’ 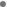.

**FIGURE 5:**
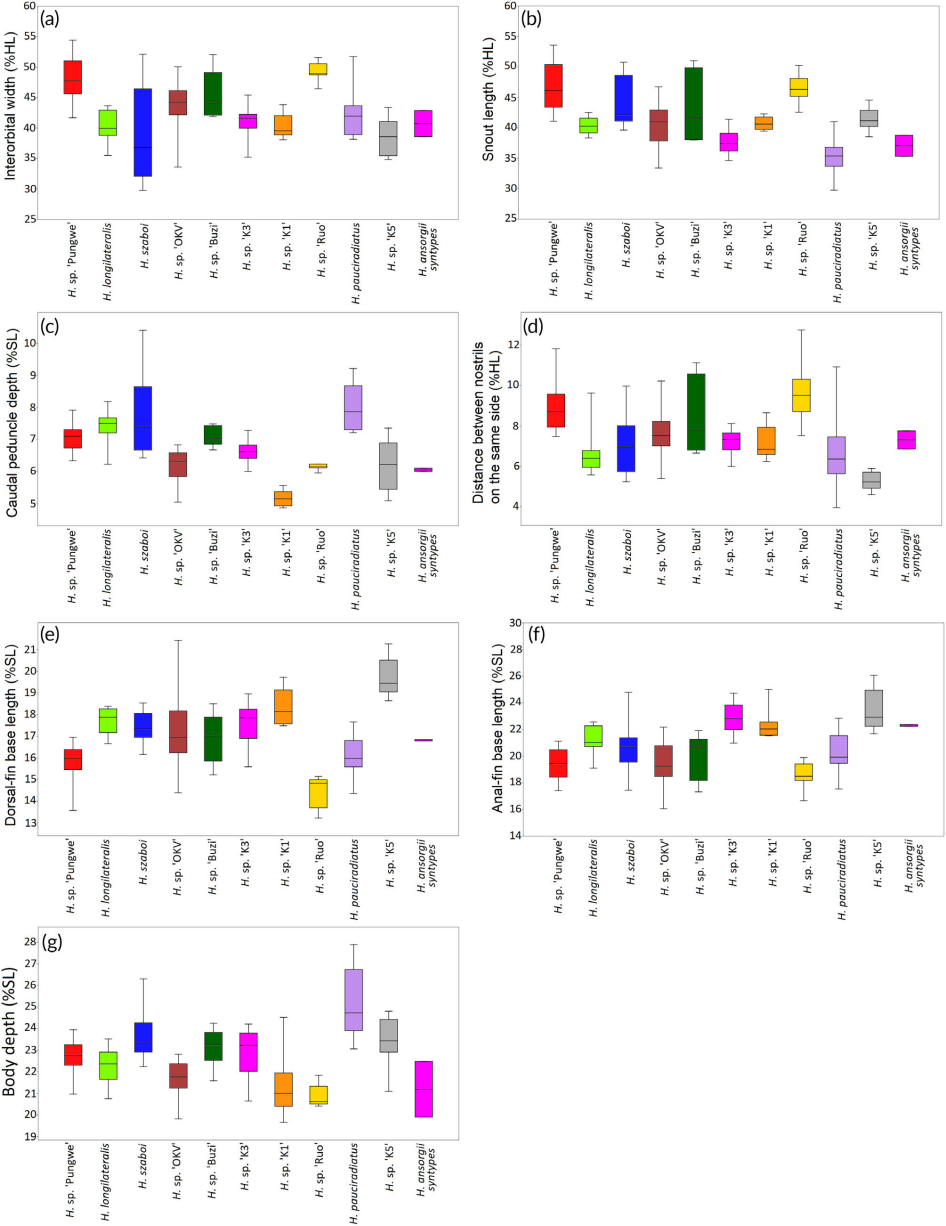
Boxplots of the morphometric characters that distinguished some of the *Heteromormyrus* lineages and species from southern Africa; the colours are consistent for each lineage or species.

Principal component analysis of the morphometric characters showed that the *Heteromormyrus* species largely overlapped. The *H. ansorgii* syntypes were negatively associated with PCI separating them from *Heteromormyrus* sp. ‘Ruo’, *Heteromormyrus* sp. ‘Pungwe’ and *Heteromormyrus* sp. ‘Buzi’, which were positively associated with this axis (Figure 4). The PCI accounted for 40.8% of the observed variance, and it was positively associated with the interorbital width (Table 2). Consistent with the pattern shown by PCI, the *H. ansorgii* syntypes (28.6%–32.9%SL) have a narrower interorbital width compared to *Heteromormyrus* sp. ‘Ruo’ (46.4%–51.6%SL), *Heteromormyrus* sp. ‘Pungwe’ (41.7%–54.4%SL) and *Heteromormyrus* sp. ‘Buzi’ (41.9%–52%SL, Figure 5a).

**TABLE 2:** Principal component loadings of the first and second axes of morphometric data from *Heteromormyrus* species and lineages Southern Africa.

Principal component	1	2
Eigenvalue	55.91	21.59
% variance	40.79	15.75
Pre‐dorsal length (PDL)	0.05	0.28
Pre‐anal length (SAF)	0.07	0.28
Pre‐pelvic length (SPF)	0.12	0.35
Pre‐pectoral length (PFS)	0.09	0.2
Pectoral fin to anal fin (PA)	−0.03	0.06
Pelvic fin to anal fin (PFA)	−0.05	−0.05
Pectoral fin to dorsal fin (PFD)	−0.01	0.16
Dorsal fin to pelvic fin (DFP)	−0.04	0.15
Dorsal‐fin base length (LD)	−0.11	−0.14
Anal‐fin base length (LA)	−0.16	−0.18
Dorsal fin to caudal fin (pD)	−0.08	−0.2
Caudal peduncle length (CPL)	0.08	−0.12
Caudal peduncle depth (CPD)	0.01	0.11
Pectoral‐fin length (LPF)	0.01	0.01
Pectoral fin to pelvic fin (PPF)	0.04	0.15
Body depth (BD)	−0.03	0.15
Mid body depth (mBD)	0.02	0.21
Head length (HL)	−0.03	0.19
Length of the snout (LSo)	0.42	−0.58
Distance between nostrils on the same side (Na)	0.14	−0.02
Orbit diameter (OD)	−0.02	−0.15
Head width (HW)	0.49	0.14
Distance between anterior nostrils (IN)	0.32	0.05
Interorbital width (IOW)	0.61	0.03

The PCII accounted for 15.8% of the observed variance, and it was positively associated with the snout length (Table 2). There was minimal separation of the species along PCII; as a result snout length could only marginally separate the species examined in the study (Figure 4). The *H. ansorgii* syntypes (35.3%–38.8%HL) are distinguished by a shorter snout than *H*. szaboi (39.6%–50.8%HL), *Heteromormyrus* sp. ‘K1’ (39.5%–42.3%HL), *Heteromormyrus* sp. ‘Ruo’ (42.6%–50.2%HL) and *Heteromormyrus* sp. ‘Pungwe’ (41.1%–53.5%HL, Figure 5b).

Further comparison of morphometric characters, including caudal peduncle depth, distance between the nostrils on the same side, dorsal‐fin base length, anal‐fin base length and body depth, revealed that they were informative in separating the different species (Figure 5). The caudal peduncles of the *H. ansorgii* syntypes (6.0%*–*6.1%SL) are narrower than *H. pauciradiatus* (7.3%*–*9.2%SL), *H. longilateralis* (7.0%–8.2%SL), *H. szaboi* (6.4%–10.4%SL), *Heteromormyrus* sp. ‘Pungwe’ (6.3%–7.9%SL) and *Heteromormyrus* sp. ‘Buzi’ (6.7%–7.5%SL), while being deeper than *Heteromormyrus* sp. ‘K1’ (4.9%–5.6%SL, Figure 5c). The distance between nostrils on the same side distinguishes the *H. ansorgii* syntypes (6.8%–7.8%HL) from *Heteromormyrus* sp. ‘K5’ (4.6%–5.9%HL, Figure 5d). The dorsal‐fin base length of *H. ansorgii* syntypes (16.8%–16.9%SL) is shorter compared to *H. longilateralis* (17.2%–18.4%SL), *Heteromormyrus* sp. ‘K5’ (18.6%–21.3%SL) and *Heteromormyrus* sp. ‘K1’ (17.5%–19.7%SL), while being longer than that of *Heteromormyrus* sp. ‘Ruo’ (13.2%–15.1%SL, Figure 5e). The *H. ansorgii* syntypes (22.2%–22.4%SL) are distinguished by a shorter anal‐fin base length compared to *Heteromormyrus* sp. ‘Pungwe’ (17.4%–21.1%SL), *Heteromormyrus* sp. ‘Buzi’ (17.3%–21.9%SL) and *Heteromormyrus* sp. ‘Ruo’ (16.6%–19.9%SL, Figure 5f). A deeper body distinguishes *H. pauciradiatus* (23.1%–27.9%SL) from the *H. ansorgii* syntypes (19.9%–22.5%SL, Figure 5g).

### 3.3 Head shape, nostril position and body colouration

**FIGURE 6:**
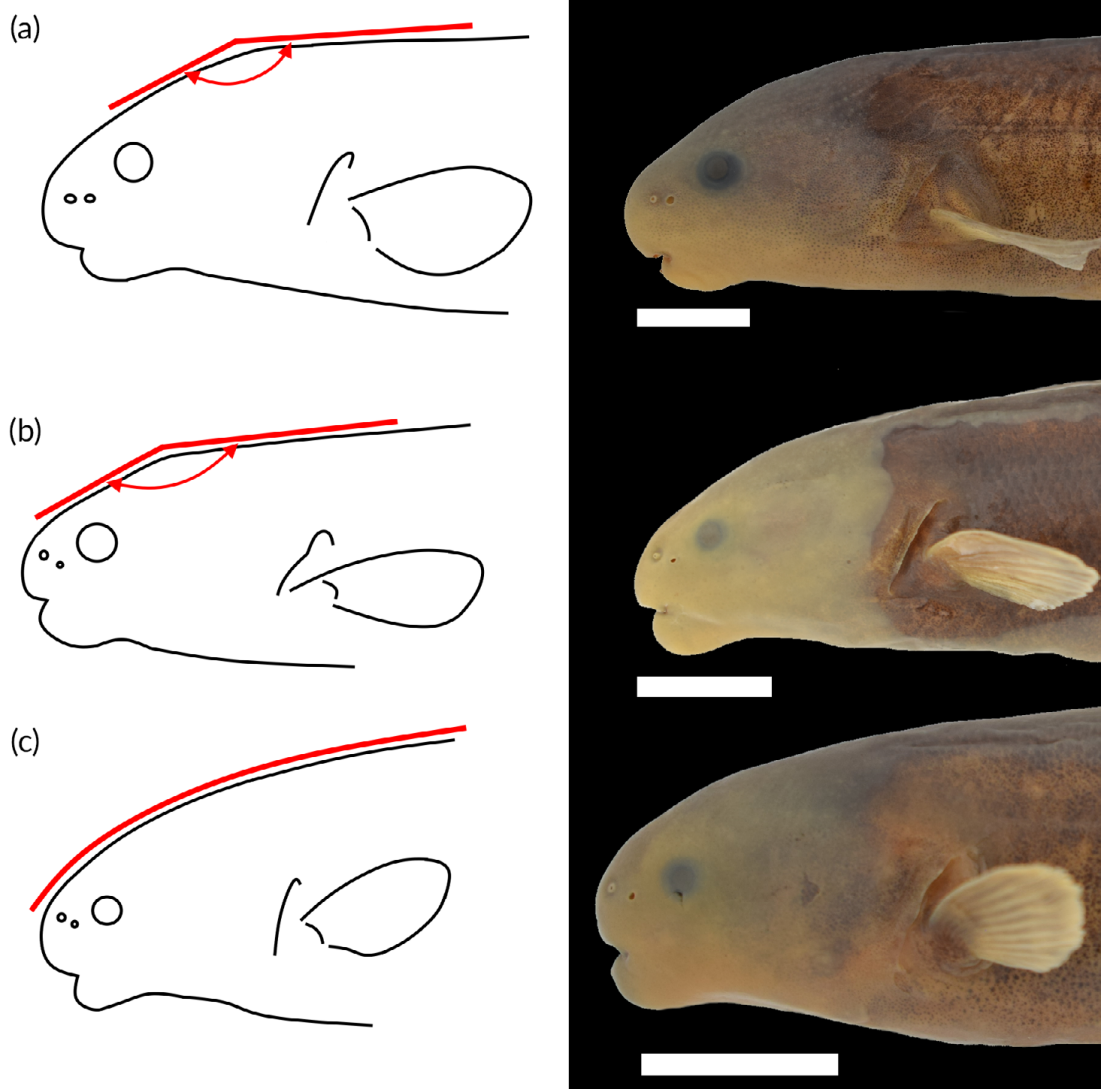
The head shapes of the *Heteromormyrus* species and lineages examined in this study. (a) *Heteromormyrus* sp. ‘K5’ (SAIAB 84645) has a straight dorsal head profile that forms an obtuse angle where it meets the rest of the dorsal body surface. The nostrils of *Heteromormyrus* sp. ‘K5’ were aligned horizontally below the level of the orbit, contrasting the other *Heteromormyrus* species/lineages, which have anterior nostrils that are always positioned higher than the posterior nostrils, and both nostrils are in line with the orbit; (b) *Heteromormyrus* sp. ‘OK’ (SAIAB 202504) similarly has a straight dorsal head profile that forms an obtuse angle where it meets the rest of the dorsal body surface; c. *Heteromormyrus* sp. ‘Pungwe’ (SAIAB 201071) has a rounded dorsal head profile that gently blends into the rest of the dorsal body surface. Scale bar = 1 cm.

The *Heteromormyrus* species are reliably separated by distinct head shapes, nostril positions and colour patterns (Figure 6). *Heteromormyrus* sp. ‘K5’ have a flat dorsal head profile that forms an obtuse angle with the rest of the dorsal body profile and a relatively large snout (Figure 6a). Similarly, *Heteromormyrus* sp. ‘OK’ and *H. longilateralis* have a flat dorsal head profile that forms an obtuse angle with the rest of the dorsal body profile and a relatively small snout (Figure 6b). *H. ansorgii*, *H. pappenheimi*, *H. tavernei*, *H. szaboi*, *Heteromormyrus* sp. ‘Ruo’, *Heteromormyrus* sp. ‘Pungwe’, *Heteromormyrus* sp. ‘Buzi’, *H. pauciradiatus* and *Heteromormyrus* sp. ‘K1’ have a rounded dorsal head profile (Figure 6c). The nostrils of *Heteromormyrus* sp. ‘K5’ were aligned horizontally below the level of the orbit. In contrast, the anterior nostrils in the rest of the *Heteromormyrus* species are always positioned higher than the posterior nostrils, and both nostrils are in line with the orbit (Figure 6).

**FIGURE 7:**
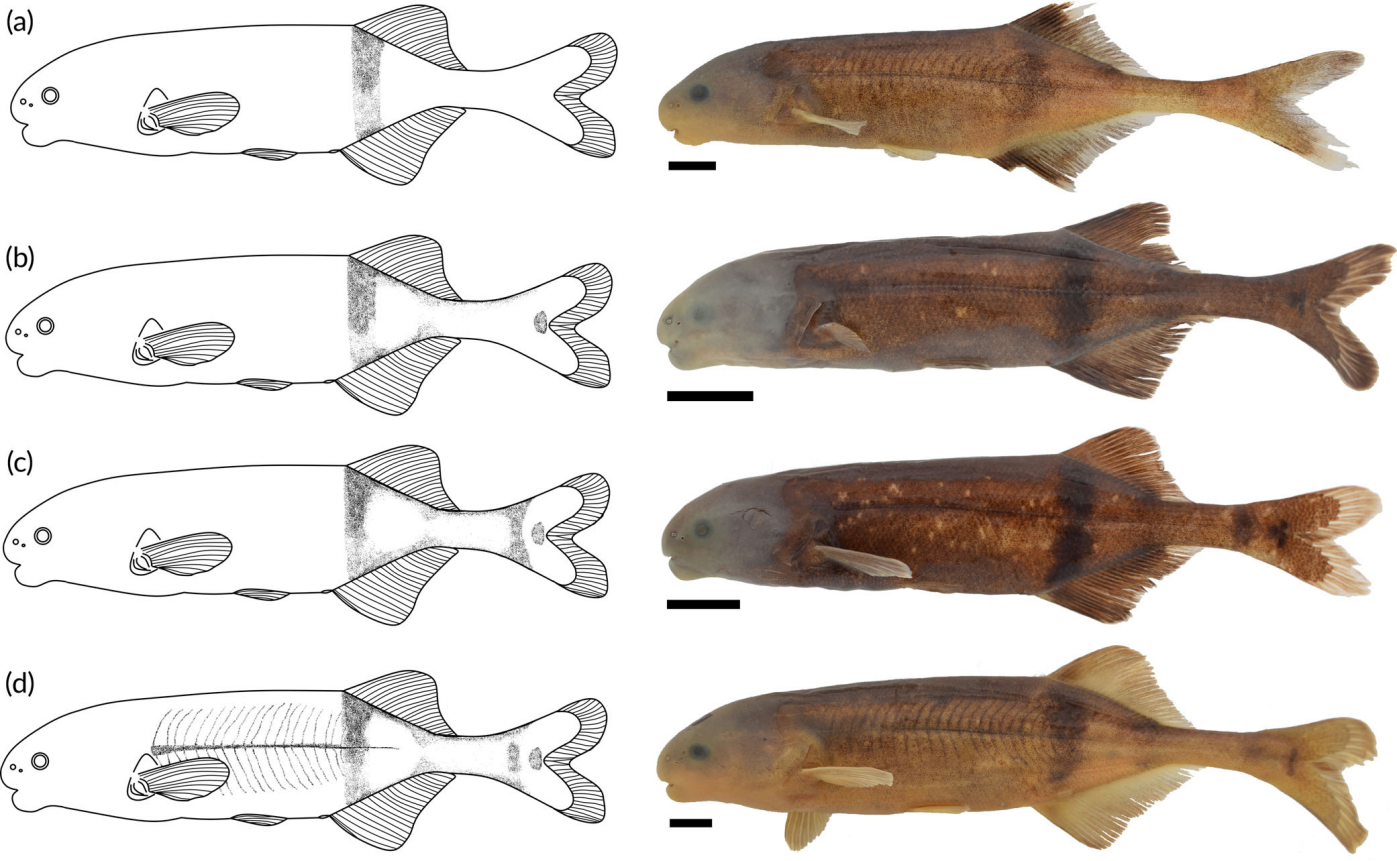
The range of colour patterns observed in *Heteromormyrus* lineages and species from southern Africa. (a) *Heteromormyrus* sp. ‘K5’ (SAIAB 84645) had barely any visible markings in the caudal peduncle. (b) *Heteromormyrus* sp. ‘K5’ (SAIAB 203161) had a clearly visible dark blotch present near the flexion point of the caudal fin. (c) *Heteromormyrus* sp. ‘K3’ (SAIAB 84790) had a clearly visible dark blotch present near the flexion point of the caudal fin and a dark vertical bar just anterior to this blotch. (d) *Heteromormyrus* sp. ‘K1’ (SAIAB 85039) had a series of clearly visible thin curved vertical bars that were more conspicuous in the anterior portion of the flank. Scale bar = 1 cm.

Live and preserved specimens examined in this study show a similar underlying colour pattern with small differences between species. The dorsal surface of all species is darker than the ventral surface. They all have a dark vertical bar beginning at the origin of the dorsal fin and ending at the anal fin. There is a dark blotch present near the flexion point of the caudal fin, and in some species, there are additional dark vertical bars in the caudal peduncle. The species are separated into four groups based on the visibility of the markings in the caudal peduncle. The first group consists of species with barely any visible markings in the caudal peduncle that included *Heteromormyrus* sp. ‘K5’, *Heteromormyrus* sp. ‘Ruo’ and *Heteromormyrus* sp. ‘Buzi’ (Figure 7a). The second group consists of *H. szaboi*, *H. longilateralis*, *Heteromormyrus* sp. ‘Pungwe’ and *Heteromormyrus* sp. ‘OK’ that have a clearly visible dark blotch present near the flexion point of the caudal fin (Figure 7b). The third group consists of *H. ansorgii*, *H. pauciradiatus* and *Heteromormyrus* sp. ‘K1’; these species have a clearly visible dark blotch present near the flexion point of the caudal fin and a dark vertical bar just anterior of this blotch (Figure 7c). The fourth group includes specimens of *Heteromormyrus* sp. ‘K1’ that have a series of clearly visible thin curved vertical bars due to the presence of melanophores within the tissue over the vertical myosepta that were more conspicuous in the anterior portion of flank (Figure 7d). Similar thin curved vertical bars were also present in some *H. longilateralis*, *Heteromormyrus* sp. ‘K5’ and *Heteromormyrus* sp. ‘OK’ specimens although they were much less visible.

### 3.4 Summary of meristic and morphological data

The meristic data show that the *H*. *ansorgii* syntypes can be separated from all the species examined, except for *H. pauciradiatus* and *Heteromormyrus* sp. ‘K3’, using a combination of characters, including total number of vertebrae, number of scales around the caudal peduncle, number of dorsal‐fin rays and the number of anal‐fin rays between the leading ray and the one directly below the leading dorsal‐fin ray. The syntypes of *H. pappenheimi* can be separated from all the species, except for *Heteromormyrus* sp. ‘K3’ and *Heteromormyrus* sp. ‘K5’, based on the same meristic characters. Although no specimens of *H. tavernei* were examined in this study, Poll's (1972) original description of this species indicates that differences in the number of scales around the caudal peduncle (20–22) and the number of anal‐fin rays (22–23) distinguish this species from its congeners, except for *H. longilateralis*, *Heteromormyrus* sp. ‘OK’ and *Heteromormyrus* sp. ‘Pungwe’.

The morphometric data from species examined in this study show that the *H*. *ansorgii* syntypes can be separated from all the species examined, except *Heteromormyrus* sp. ‘K3’, using a combination of characters, including interorbital width, snout length, caudal peduncle depth, distance between the nostrils on the same side, dorsal‐fin base length, anal‐fin base length and body depth. The measurements of *H. tavernei* provided in Poll's (1972) original description suggest that it can be distinguished from all the species examined in this study by the combination of the interorbital width (24.7%–30.0%HL), snout length (24.7%–30.0%HL), caudal peduncle depth (6.9%–8.6%SL) and head length (25.8%–29.2%SL). Similarly, Boulenger's (1910) original description of *H. pappenheimi* suggests that this species has a deeper body (25.0%–27.3%TL) compared to *Heteromormyrus* sp. ‘K3’ (18.8%–21.5%TL) and *Heteromormyrus* sp. ‘K5’ (18.3%–21.7%TL).

Using a combination of meristic characters, morphometric characters, head shapes, nostril positions and variation in colour pattern, the present study was able to distinguish the *H. ansorgii* syntypes from the other two previously described species in the Kwanza River system, *H. pauciradiatus*, *H. pappenheimi*, as well as two candidate species, *Heteromormyrus* sp. ‘K1’ and *Heteromormyrus* sp. ‘K5’. However, the *H. ansorgii* syntypes cannot be separated from one of the candidate species in this system, *Heteromormyrus* sp. ‘K3’, based on any of these characters. Our results also show that the *Heteromormyrus* sp. ‘K3’ lineage is distinct from both *H. pauciradiatus* and *H. pappenheimi*. Based on these findings, we propose that the *Heteromormyrus* sp. ‘K3’ lineage is likely to be conspecific with *H. ansorgii* s.s. This species is herein redescribed based on two syntypes and 15 specimens (previously designated as *Heteromormyrus* sp. ‘K3’) collected between 2007 and 2008 from the Kwanza River system. *H. pauciradiatus*, a species that was described based on a specimen with a deformed caudal peduncle (Sullivan et al., 2022), is also redescribed in this study using 22 specimens collected between 2007 and 2008. None of the specimens examined in this study were morphologically consistent with *H. pappenheimi*. Based on consistent meristic and morphometric differences, six new species are described in the present study: *Heteromormyrus* sp. ‘K5’ as *Heteromormyrus dolichorhynchus* sp. nov., *Heteromormyrus* sp. ‘K1’ as *Heteromormyrus angusticaudata* sp. nov., *Heteromormyrus* sp. ‘OK’ as *Heteromormyrus xanekweorum* sp. nov., *Heteromormyrus* sp. ‘Ruo’ as *Heteromormyrus chilembwei* sp. nov., *Heteromormyrus* sp. ‘Pungwe’ as *Heteromormyrus tangwenai* sp. nov. and *Heteromormyrus* sp. ‘Buzi’ as *Heteromormyrus ndauorum* sp. nov.

## 4 TAXONOMIC ACCOUNTS

### 4.1 *H. ansorgii* (Boulenger 1905)

See Figure 8.

**FIGURE 8:**
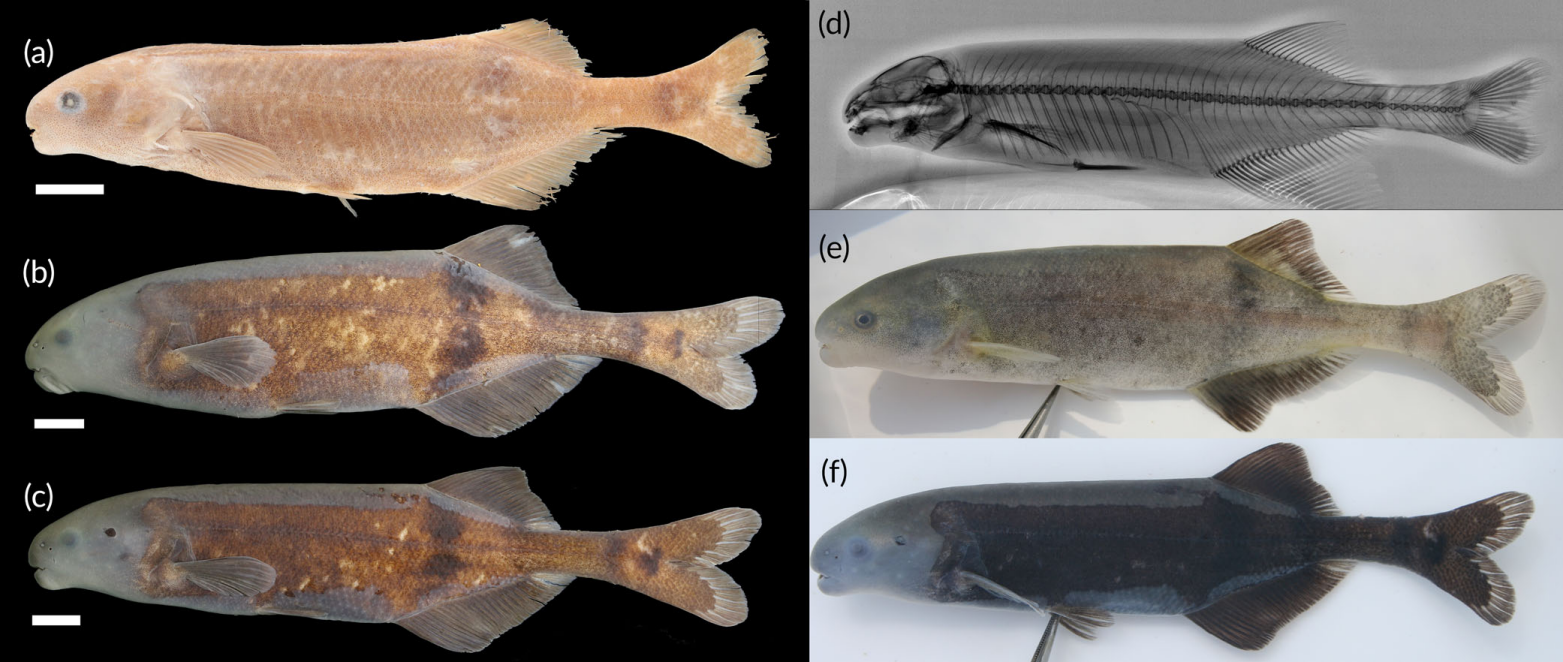
(a) Syntype of *Heteromormyrus ansorgii* 100.1 (BMNH 1905.5.29.62) mm standard length (SL), female, from between Benguella and Bihé, Angola, collected by WJ Ansorge. (Picture provided by Luis Moreira da Costa); (b) recently collected *H. ansorgii* female specimen 110.0 mm SL (SAIAB 84790) from the Kwanza River, Angola, southern Africa; (c) recently collected *H. ansorgii* male specimen 120.0 mm SL (SAIAB 84790) from the Kwanza River; (d) X‐ray radiograph of syntype; (e, f) live pictures of *H. ansorgii*. Scale bar =1 cm.

*Marcusenius ansorgii* Boulenger, 1905.

*Hippopotamyrus ansorgii* (Boulenger, 1905) new combination by Taverne, 1971.

*H. ansorgii* (Boulenger, 1905) new combination by Sullivan et al., 2022.

*Heteromormyrus* sp. ‘K3’ in Mutizwa et al. (2021).

#### 4.1.1 Syntypes

BMNH 1905.5.29.62100.1 mm SL, between Benguella and Bihé, Angola, W. J. Ansorge. BMNH 1905.5.29.63, 98.5 mm SL, between Benguella and Bihé, Angola, W. J. Ansorge. Mutizwa et al. (2021) proposed that these specimens were likely collected from the Kwanza River system.

#### 4.1.2 Non‐type specimens

SAIAB 84763, 1 specimen, 61.1 mm SL, Kwanza River at confluence with Kawa River, 9° 10′ 14″ S, 13° 21′ 59″ E, Kwanza River system, Angola, E. Swartz and A. Chakona, 18 October 2007, GenBank genseq‐3 COI: MW600862, S7 intron: MW756326, MW756327; SAIAB 85028, 3 specimens, 80.6*–*89.1 mm SL, bridge on road east of Camacupa, Kwiva River, 11° 59′ 3″ S, 17° 43′ 21″ E, Kwanza River system, Angola, E. Swartz, 10 August 2008, BOLD genseq‐3 COI: SAFW466‐09, SAFW467‐09; SAIAB 85177; 3 specimens 79.1*–*142.6 mm SL, bridge between Cimanga and Capunda, Luando River, 10° 38′ 26″ S, 17° 25′ 6″ E, Kwanza River system, Angola, E. Swartz and A. Chakona, 23 August 2008, BOLD genseq‐3 COI: SAFW572‐09, SAFW577‐09, SAFW566‐09; SAIAB 84636, 4 specimens, 94.1*–*112 mm SL, Terra Nova, 09° 46′ 44″ S, 14° 31′ 24″ E, Kwanza River system, Angola, E. Swartz and A. Chakona, 9 October 2007, BOLD genseq‐3 COI: SAFW252‐08, SAFW250‐08; SAIAB 84790, 5 specimens 59.5*–*138.6 mm SL, Posto 5, 09° 48′ 23″ S, 15° 24′ 30″ E, Kwanza River, Angola, E. Swartz and A. Chakona, 24 October 2007, GenBank genseq‐3 COI: MW600861.

#### 4.1.3 Diagnosis

*H. ansorgii* has 14–16 scales around the caudal peduncle, distinguishing it from *H. tavernei* (20–22), *H. longilateralis* (20) and *H. tangwenai* sp. nov. (18*–*20). The number of anal‐fin rays between the leading ray and the one directly below the leading dorsal‐fin ray distinguishes *H. ansorgii* (6–9) from *H. longilateralis* (3–5), *H. szaboi* (3–4), *H. xanekweorum* sp. nov. (4–5) and *H. ndauorum* (3–4). Fewer dorsal‐fin rays distinguish *H. ansorgii* (18*–*21) from *H. angusticaudata* sp. nov. (22*–*24) and *H. dolichorhynchus* sp. nov. (22*–*25). *H. ansorgii* is further distinguished from *H. angusticaudata* sp. nov. by having fewer total vertebrae 42*–*44 (vs. 46*–*47), fewer caudal vertebrae 24–25 (vs. 27–27) and fewer anal‐fin rays 23*–*27 (vs. 28*–*30). *H. ansorgii* is distinguished from *H. chilembwei* sp. nov. by having fewer scales along the caudal peduncle 16*–*19 (vs. 21*–*23) and fewer scales along the lateral line 68*–*77 (vs 82*–*84). *H. ansorgii* is distinguished from *H. pauciradiatus* by a slender caudal peduncle 6.0%*–*7.2%SL (vs. 7.3%*–*9.3%SL). A narrower body distinguishes *H*. *ansorgii* (18.8%–21.5%TL) from *H. pappenheimi* (25.0%–27.3%TL). Interorbital width further distinguishes *H. ansorgii* (28.6%–45.4%HL) from *H. chilembwei* sp. nov. (46.4%–51.6%HL). Snout length distinguishes *H. ansorgii* (34.6%–41.4%HL) from *H. tavernei* (24.7%–30.0%HL) and *H. chilembwei* sp. nov. (42.6%–50.2%HL). Head length further distinguishes *H. ansorgii* (19.4%–24.9%SL) from *H. tavernei* (25.8%–29.2%SL). *H. ansorgii* is further distinguished from *H. szaboi* by a greater distance between pectoral and dorsal fins 41.6%*–*46.0%SL (vs. 38.9%*–*41.5%SL). Presence of a clearly visible dark blotch near flexion point of the caudal fin and a dark vertical bar on the caudal peduncle distinguishes *H. ansorgii* from *H. dolichorhynchus* sp. nov., *H. chilembwei* sp. nov. and *H. ndauorum* sp. nov. that lack any clearly visible marks on caudal peduncle; *H. longilateralis*, *H. szaboi*, *H. tangwenai* sp. nov. and *H. xanekweorum* sp. nov. have only a dark blotch near flexion point of the caudal fin.

#### 4.1.4 Description

Morphometric proportions and meristics are summarised in Table 3, with meristic counts of the syntypes given in parentheses.

**TABLE 3:** Proportional measurements and meristic data for *Heteromormyrus* examined in this study.

	*Heteromormyrus ansorgii* syntype	*H. ansorgii* syntype	*H. ansorgii* (*H*. Sp. ‘K3’)	*Heteromormyrus pappenheimi*	*Heteromormyrus tavernei*	*Heteromormyrus pauciradiatus*
Number of specimens			15	8	12	22
Total length	113.2	113.5	66.7–167.5 (109.4 ± 26.4)	–	–	40.0–135.1 (78.8 ± 28.1)
Standard length	98.5	100.1	59.5–142.6 (96.8 ± 22.6)	80–180*	53–139*	36.3–118.7 (69.4 ± 24.8)
Head length	24.5	21.9	12.8–29.4 (21 ± 4.3)	–	–	9.5–27.2 (17.2 ± 5.5)
Pre‐dorsal length	63.8	68.1	62.1–67.0 (64.9 ± 1.4)	–	41.3–47.0*	64.0–68.8 (65.9 ± 1.4)
Pre‐anal length	60.7	65.1	56.6–61.0 (59.3 ± 2)	–	18.0–22.0*	58.4–63.6 (61.1 ± 1.6)
Pre‐pelvic length	40.4	43	35.6–40.7 (38.9 ± 1.7)	–	–	39.4–45.6 (42.6 ± 2)
Pre‐pectoral length	24.6	23	21.8–25.3 (23.5 ± 1)	–	–	24.1–29.1 (26.7 ± 1.3)
Pectoral fin to anal fin	35.9	39.8	33.2–38.4 (36.3 ± 1.6)	–	–	32.9–40.9 (35.4 ± 1.5)
Pelvic fin to anal fin	20	20.7	17.3–21.8 (20.2 ± 1)	–	–	16.5–21.5 (18.8 ± 1.3)
Pectoral fin to dorsal fin	42.2	46	41.6–45.4 (43.4 ± 1.3)	–	–	39.0–44.6 (42.5 ± 1.4)
Dorsal fin to pelvic fin	31.3	33.7	31.4–36.0 (33.4 ± 1.2)	–	–	30.4–37.3 (34.1 ± 1.8)
Dorsal‐fin base length	16.9	16.8	15.6–19.0 (17.4 ± 1)	–	15.2–19.1*	14.4–17.7 (16.4 ± 1.5)
Anal‐fin base length	22.2	22.4	21.0–24.7 (22.8 ± 1.1)	–	19.3–22.3*	17.5–22.8 (20.1 ± 1.5)
Dorsal fin to caudal fin	38.8	38.8	36.8–40.3 (38.8 ± 1.1)	–	–	34.9–40.2 (38.1 ± 1.6)
Caudal peduncle length	18.7	16.2	18.2–21.4 (19.4 ± 1.2)	–	16.4–21.1*	17.6–23.8 (20.4 ± 1.4)
Caudal peduncle depth	6	6.1	6.0–7.2 (6.6 ± 0.4)	–	6.9–8.6*	7.3–9.2 (8.1 ± 0.7)
Pectoral‐fin length	16.2	16.2	14.5–18.0 (16.2 ± 1)	–	17.2–20.9*	16.6–21.5 (18.6 ± 1.3)
Pectoral fin to pelvic fin	15.9	17.3	14.7–18.0 (16.6 ± 0.9)	–	–	16.1–19.6 (17.8 ± 1.1)
Body depth	19.9	22.5	20.6–24.2 (22.8 ± 1.2)	–	22.2–24.2*	23.1–27.9 (25.4 ± 1.6)
Mid body depth	20.4	20.9	18.7–23.4 (21.3 ± 1.4)	–	–	22.5–27.3 (24.9 ± 2.1)
Head length	24.9	21.9	19.4–23.6 (21.9 ± 1.3)	–	25.8–29.2*	22.9–27.5 (25.1 ± 1.3)
Length of the snout	35.3	38.8	34.6–41.4 (36.5 ± 4.1)	–	24.7–30.0*	29.7–41.0 (35.5 ± 2.6)
Distance between nostrils on the same side	7.8	6.8	6.0–8.1 (7.2 ± 0.6)	–	–	3.9–10.9 (6.5 ± 1.6)
Orbit diameter	15.9	17.4	7.7–20.3 (13 ± 3.2)	–	12.4–16.7*	9.4–16.5 (12.6 ± 1.9)
Head width	47.8	50.7	47.5–61.4 (54.1 ± 3.4)	–	–	50.1–61.2 (55.9 ± 2.7)
Distance between anterior nostrils	24.7	22.8	21.0–30.5 (24.7 ± 2.3)	–	–	23.4–31.0 (26.1 ± 1.9)
Interorbital width	28.6	32.9	35.2–45.4 (40.7 ± 2.7)	–	24.7–30.0*	38.2–51.7 (42 ± 3)
Counts						
Number of scales around caudal peduncle	16	16	16 (14–16)	12–14*	20–22*	16 (16–18)
Number of scales along caudal peduncle	17	16	18 (17–19)	–	–	16 (14–18)
Number of scales along lateral line	70	69	72 (68–77)	71–80*	70–77*	70 (60–74)
Teeth on upper jaw	7	5	7 (6–8)	5–7*	6–10*	7 (6–7)
Teeth on lower jaw	4	9	8 (7–9)	6–8*	8–10*	8 (6–9)
Number of pectoral‐fin rays	10	9	10 (9–11)	–	–	10 (9–11)
Number of pelvic‐fin rays	6	5	6 (6–6)	–	–	6
X‐ray counts						
Number of specimens	_	_	8	8	–	7
First anal‐fin ray to first dorsal‐fin ray	6	6	7 (6–9)	9 (6–9)	–	7 (6–9)
Total vertebrae	42	43	43 (42–44)	42 (42–43)	–	41 (40–42)
Pre‐caudal vertebrae	18	18	18 (18–19)	18 (17–18)	–	18 (18–19)
Caudal vertebrae	24	25	25 (24–25)	24 (24–25)	–	23 (22–24)
Vertebrae before the first dorsal‐fin radial	20	21	21 (21–22)	19 (19–20)	–	20 (20–21)
Vertebrae before the first anal‐fin radial	19	18	19 (19–20)	18	–	18 (18–19)
Dorsal‐fin rays	18	18	20 (18–21)	22 (21–23)	17–20*	20 (18–21)
Anal‐fin rays	24	26	25 (23–27)	27 (26–28)	22–23*	23 (23–26)

*Note*: The ranges of the measurements are presented with the mean and standard deviation inside the parentheses. For the counts, the number next to the parentheses represents the mode, and the numbers in the parenthesis represent the range. The asterisks (*) indicates the values obtained from the original description of the species.

Rounded blunt snout, below eye level. Dorsal profile of head convex from snout to back of the head where it becomes gently inclined towards dorsal fin. Small sub‐terminal mouth in line with pectoral‐fin base. Small rounded chin swelling transitions into concave ventral profile of head. Round orbit. Anterior and posterior nostrils laterally positioned, closer to snout tip than opercular opening, anterior to and arranged horizontally in line with orbit. Anterior nostril positioned slightly higher than posterior nostril. Small gill opening with soft skin cover adjacent to pectoral‐fin base. Bicuspid teeth 5–8 (7) in upper jaw and 4–9 (4) in lower jaw.

Laterally compressed body with greatest width between gill covers. Slender fusiform body with greatest depth occurring between origin of dorsal and anal fins. Body tapers gently towards head but sharply from origins of both dorsal and anal fins to caudal fin. Caudal peduncle slender, same depth from its origin to roughly around half its length, gradually widening into two symmetrical lobes. Body covered with transparent membrane that becomes increasingly translucent to opaque towards head, dorsal and ventral surfaces in preserved specimens. Head without scales; rest of the body covered in small cycloid scales with reticulated striae. Lateral line originates above pectoral fin and extends to caudal peduncle. There are 68*–*77 (70) scales along lateral line, 16*–*19 (17) of them along caudal peduncle length and 14–16 (16) around caudal peduncle. Urogenital opening situated just anterior to origin of anal fin.

Elliptical pectoral fin with 9–11 (10) rays, extends just beyond origin of pelvic fin. Short pelvic fin with 6 (6) rays. The first two dorsal‐ and anal‐fin rays are unbranched, unsegmented and small. These two unbranched and unsegmented rays are followed by a single long unbranched and segmented ray, followed by numerous branched, segmented rays. The last dorsal and anal ray is usually branched all the way to its base. The first two dorsal‐ and anal‐fin rays are unbranched, unsegmented and small. Anal and dorsal fins set towards posterior of body. The last dorsal and anal ray is usually branched all the way to its base. Anal‐fin origin 6–9 (6) fin rays anterior to dorsal‐fin origin. Dorsal‐fin rays: 18*–*21 (18); anal‐fin rays: 23*–*27 (24). Caudal fin deeply forked with rounded lobes, with bases covered in scales; distance from caudal‐fin flexion point to caudal‐fin tips is roughly equal to caudal peduncle length.

See Figure 8. Live colour: body colour ranges from silver with a tinge of brown to a very dark brown. Dorsal surface usually darker than the ventral surface. Dark vertical bar originates from origin of dorsal fin. Dark blotch presents near the flexion point of the caudal fin and a dark vertical bar just anterior to this blotch. Fins brown, darker than the body in silver specimens.

Preserved in ethanol: body colour ranges from light to dark brown. Dorsal surface usually darker than the ventral surface. Dark vertical bar originates from origin of dorsal fin. Clearly visible dark blotch presents near the flexion point of the caudal fin and a dark vertical bar just anterior of this blotch. Fins brown like the body.

Determining the sex of adults can be done externally by examining their anal‐fin base. In males, the body wall is dorsally indented giving the dorsal margin of the anal fin a sigmoid curvature. In females, the body wall appears almost straight.

Total vertebrae: 42*–*44 (42), pre‐caudal vertebrae: 18–19 (18), caudal vertebrae: 24–25 (24), number of vertebrae at first dorsal radial: 20*–*22 (20), number of vertebrae at first anal radial: 18*–*20 (18).

#### 4.1.5 Distribution

**FIGURE 9:**
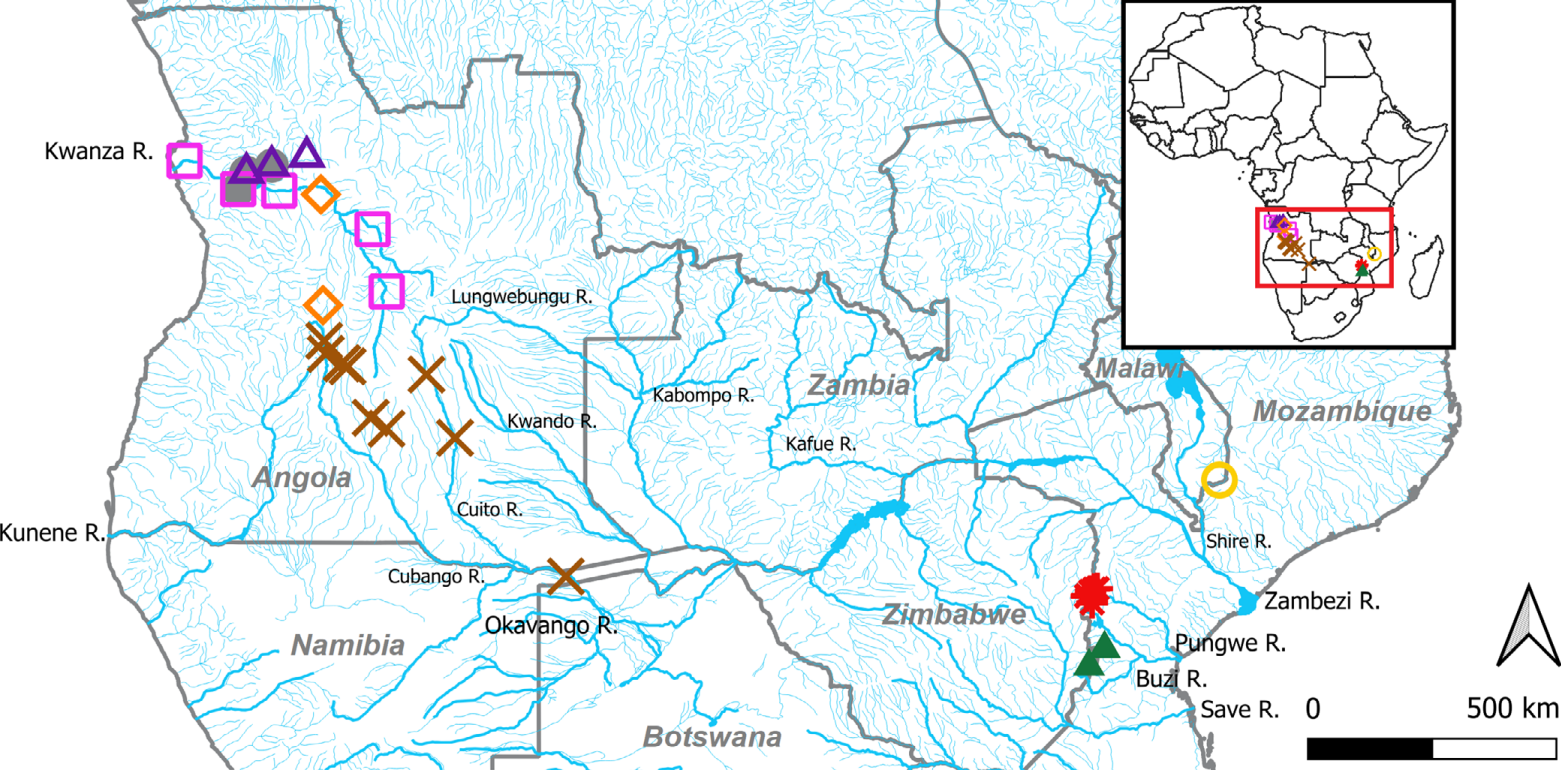
Distribution of the *Heteromormyrus* species described in this study. *Heteromormyrus ansorgii* 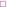, *Heteromormyrus pauciradiatus* 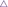, *Heteromormyrus angusticaudata* sp. nov.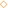 and *Heteromormyrus dolichorhynchus* sp. nov.  are restricted to the Kwanza River. *Heteromormyrus xanekweorum* sp. nov.  is restricted to the Okavango River. *Heteromormyrus chilembwei* sp. nov.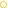 is found in the Ruo River. *Heteromormyrus tangwenai* sp. nov.  is endemic to the Pungwe River. *Heteromormyrus ndauorum* sp. nov. 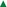 is endemic to the Buzi River.

*H. ansorgii* is known from the lower, middle and upper sections of the Kwanza River mainstem and its tributary, the Lwando River, Angola (Figure 9). However, historical connectivity of these populations is likely to have been fragmented by the construction of three major hydroelectric dams, the Cambambe, Lauca and Capanda dams on the mainstem Kwanza River.

#### 4.1.6 Etymology

The species was named in honour of Dr. William John Ansorge, who made extensive collections of various animals, which include the syntypes of *H. ansorgii* from the Kwanza River system during his expedition in Angola from 1903 to 1909.

### 4.2 *H. pauciradiatus* Steindachner 1866

See Figure 10.

**FIGURE 10:**
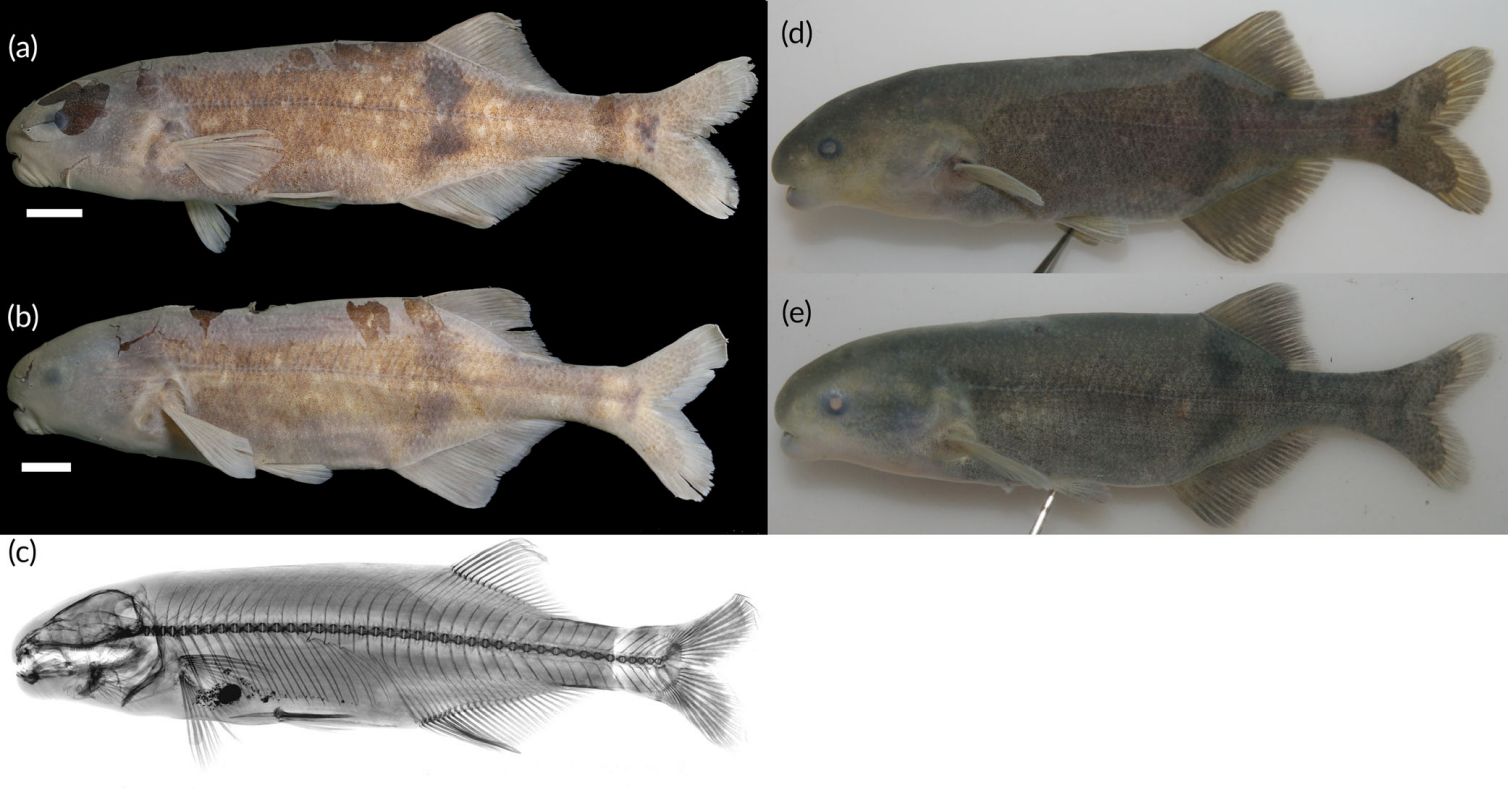
(a) *Heteromormyrus pauciradiatus* (SAIAB 85209), female 118.7 mm standard length (SL), from the Kwanza River; (b) *H. pauciradiatus* (SAIAB 85203), male 108.7 mm SL, from the Kwanza River; (c) X‐ray radiograph of holotype; (d, e) live pictures of *H. pauciradiatus*. Scale bar =1 cm.

*Mormyrus* (*Heteromormyrus*) *pauciradiatus* Steindachner, 1866.

*Marcusenius pauciradiatus* (Steindachner, 1866) new combination by Boulenger, 1898.

*M*. (*Heteromormyrus*) *pauciradiatus* (Steindachner, 1866) new subgeneric combination by Géry, 1968.

*Pollimyrus pauciradiatus* (Steindachner, 1866) new combination by Taverne, 1971.

*Heteromormyrus pauciradiatus* (Steindachner, 1866) new combination by Taverne, 1972.

*Hippopotamyrus* sp. ‘K4’ in Mutizwa et al. (2021).

#### 4.2.1 Holotype

NMW 22417 lodged in the Naturhistorisches Museum Vienna, 100 mm TL, type locality only given as ‘Angola’, GenBank genseq‐1 mitogenome: ON533765.

#### 4.2.2 Non‐type specimens

SAIAB 85209, 2 specimens, 88.0–115.0 mm SL, farm below Lucala 2 bridge, 9° 25′ 30″ S, 14° 42′ 0″ E, Kwanza River system, Angola, E. Swartz and A. Chakona, 25 August 2008, BOLD genseq‐3 COI: SAFW619‐09, GenBank genseq‐3 cyt *b*: MW600880, S7 intron: MW756330, MW756331; SAIAB 85203, 2 specimens 94.0–176.0 mm SL, farm below Lucala 2 bridge, 9° 25′ 30″ S, 14° 42′ 0″ E, Kwanza River system, Angola, E. Swartz and A. Chakona, 25 August 2008, BOLD genseq‐3 COI: SAFW599‐09, SAFW596‐09, GenBank genseq‐3 cyt *b*: MW600881, S7 intron: MW756368, MW756369; SAIAB 84683, 4 specimens 54.7–86.2 mm SL, N'dalatando farm, Lucala River, Kwanza River system, 9° 25′ 30″ S, 14° 42′ 0″ E, Kwanza River system, Angola, E. Swartz and A. Chakona, 11 October 2007, BOLD genseq‐3 COI: SAFW274‐08; SAIAB 85102, 1 specimen 79.2 mm SL, Lucala River bridge, 9° 16′ 8″ S, 15° 14′ 49″ E, Kwanza River system, Angola, E. Swartz and A. Chakona, 18 August 2008, BOLD genseq‐3 COI: SAFW493‐09; SAIAB 85120, 16 specimens, 36.3–94.4 mm SL, above Calandula Falls immediately above the major waterfall, Lucala River, 9° 4′ 26″ S, 16° 0′ 0″ E, Kwanza River system, Angola, E. Swartz and A. Chakona, 19 August 2008, BOLD genseq‐3 COI: SAFW518‐09, SAFW515‐09; SAIAB 85139, 1 specimen 97.1 mm SL, below Calandula Falls below the major waterfall, Lucala River, 9° 4′ 37″ S, 15° 59′ 59″ E, Kwanza River system, Angola, E. Swartz and A. Chakona, 19 August 2008, BOLD genseq‐3 COI: SAFW434‐08. SAIAB 84716, 1 specimen 65.0 mm SL, confluence of Kawa and Kwanza rivers, Kawa River, 09° 10′ 17″ S, 13° 22′ 5″ E, Kwanza River system, Angola, E. Swartz, 16 August 2007, GenBank genseq‐3 COI: MW600858; SAIAB 84708, 18 specimens 64.0–135.0 mm SL, Lucala 3, Lucala River, 09° 31′ 21″ S, 14° 23′ 10″ E, Kwanza River system, Angola, E. Swartz, 12 November 2007, BOLD genseq‐3 COI: MW600856, MW600857, GenBank genseq‐3 cyt *b*: MW600882, MW600883, S7 intron: MW756366, MW756367, MW756358, MW756359.

#### 4.2.3 Diagnosis

A deep caudal peduncle distinguishes *H. pauciradiatus* (7.3%*–*9.2%SL) from *H. ansorgii* (6.0%*–*7.2%SL), *H. angusticaudata* sp. nov. (4.9%–5.6%SL), *H. xanekweorum* sp. nov. (5.0%–6.8%SL) and *H. chilembwei* sp. nov. (5.9%–6.2%SL). A deep body further separates *H. pauciradiatus* (23.1%–27.9%SL) from *H. xanekweorum* sp. nov. (19.8%–22.8%SL) and *H. chilembwei* sp. nov. (20.4%–21.8%SL). *H. pauciradiatus* has 16–18 scales around the caudal peduncle that distinguish it from *H. pappenheimi* (12–14), *H. tavernei* (20–22) and *H. longilateralis* (20). Number of anal‐fin rays between the leading ray and the one directly below the leading dorsal‐fin ray in *H. pauciradiatus* (6–9) distinguishes it from *H. longilateralis* (3–5), *H. szaboi* (3–4), *H. xanekweorum* sp. nov. (4–5) and *H. ndauorum* sp. nov. (3–4). Fewer scales along the caudal peduncle distinguish *H. pauciradiatus* (14–18) from *H. longilateralis* (19–24), *H. chilembwei* sp. nov. (21*–*23) and *H. angusticaudata* sp. nov. (19–24). Fewer dorsal‐fin rays distinguish *H. pauciradiatus* (18*–*21) from *H. angusticaudata* sp. nov. (22*–*24) and *H. dolichorhynchus* sp. nov. (22*–*25). Fewer vertebrae also separate *H. pauciradiatus* (40*–*42) from *H. angusticaudata* sp. nov. (46*–*47) and *H. chilembwei* sp. nov. (43). Fewer anal‐fin rays further distinguish *H. pauciradiatus* (23–26) from *H. angusticaudata* sp. nov. (28*–*30). Combination of a clearly visible dark blotch present near flexion point of the caudal fin and a dark vertical bar on caudal peduncle distinguishes *H. pauciradiatus* from *H. dolichorhynchus* sp. nov., *H. chilembwei* sp. nov. and *H. ndauorum* sp. nov. that do not have any clearly visible marks on caudal peduncle; *H. longilateralis*, *H. szaboi*, *H. tangwenai* sp. nov. and *H. xanekweorum* sp. nov. have only a dark blotch present near flexion point of the caudal fin.

#### 4.2.4 Description

Morphometric proportions and meristics are summarised in Table 4.

**TABLE 4:** Proportional measurements and meristic data for *Heteromormyrus* examined in this study.

	*Heteromormyrus longilateralis*	*Heteromormyrus szaboi*	*Heteromormyrus angusticaudata* sp. nov. holotype	*Heteromormyrus angusticaudata* sp. nov.	*Heteromormyrus dolichorhynchus* sp. nov. holotype	*H. dolichorhynchus* sp. nov.
Number of specimens	11	13	–	8	–	12
Total length	64.2–183.0 (141.2 ± 36.6)	46.0–148.5 (84 ± 30.9)	133.1	66.2–173.0 (121.4 ± 34.2)	173	85.0–176.0 (138.2 ± 31.7)
Standard length	57.1–162.0 (125.4 ± 32.7)	41.3–134.5 (75 ± 27.8)	118.3	59.6–152.5 (108.7 ± 29.7)	151	73.1–155.0 (120.6 ± 27.6)
Head length	13.2–36.1 (27.3 ± 6.6)	9.7–33.7 (18.8 ± 6.9)	27.3	14.3–33.2 (24.1 ± 5.8)	36.2	16.9–36.2 (26.9 ± 5.8)
Pre‐dorsal length	60.8–66.5 (63.7 ± 1.9)	62.0–66.6 (65.1 ± 0.8)	63.7	61.9–63.7 (62.5 ± 0.6)	65.3	58.9–65.3 (61.8 ± 2.1)
Pre‐anal length	59.5–62.2 (60.6 ± 0.9)	58.5–63.3 (61.8 ± 0.9)	61.7	56.8–62.6 (58.6 ± 1.7)	60.8	54.6–60.8 (57 ± 2)
Pre‐pelvic length	38.4–42.8 (40.3 ± 1.3)	40.0–45.5 (44 ± 0.9)	40.3	36.7–40.3 (38.8 ± 1.1)	40.3	35.0–40.3 (37.6 ± 1.7)
Pre‐pectoral length	22.4–26.4 (24.1 ± 1.4)	24.5–29.6 (27.3 ± 1.4)	23.3	22.4–26.2 (23.5 ± 1.3)	25	21.3–25.7 (23.7 ± 1.5)
Pectoral fin to anal fin	34.6–40.2 (38.2 ± 1.5)	34.1–38.3 (36.3 ± 1.2)	38	33.1–38.0 (35.6 ± 1.6)	36.8	33.1–36.8 (34.9 ± 1.1)
Pelvic fin to anal fin	18.2–22.5 (20.6 ± 1.1)	17.0–19.3 (18 ± 0.8)	21	17.9–21.0 (19.9 ± 1)	21	18.5–21 (19.5 ± 0.9)
Pectoral fin to dorsal fin	39.1–43.8 (41.9 ± 1.6)	38.9–41.5 (40.5 ± 0.8)	43.5	38.9–43.5 (41.4 ± 1.6)	42.3	38.4–42.4 (40.1 ± 1.2)
Dorsal fin to pelvic fin	29.7–33.2 (31.7 ± 1.1)	28.8–33.5 (31.2 ± 1)	31.7	30.5–33.8 (31.6 ± 1.1)	33.3	29.2–33.7 (31.4 ± 1.3)
Dorsal‐fin base length	17.2–18.4 (17.7 ± 0.6)	16.2–18.5 (17.4 ± 0.7)	17.6	17.5–19.7 (18.4 ± 0.9)	20.5	18.6–21.3 (19.8 ± 0.9)
Anal‐fin base length	19.1–22.6 (21.1 ± 1.1)	17.4–24.8 (20.6 ± 1.9)	21.7	21.5–25.0 (22.4 ± 1.3)	23.4	21.7–26.1 (23.6 ± 1.6)
Dorsal fin to caudal fin	35.6–42.1 (39.3 ± 1.8)	37.4–40.5 (38.7 ± 1.1)	39.4	38.3–40.9 (40.1 ± 0.9)	40.5	39.8–44.9 (42 ± 1.7)
Caudal peduncle length	19.9–23.4 (21.2 ± 0.9)	18.2–22.2 (20.2 ± 1.7)	20.4	19.2–22.7 (20.7 ± 1.1)	20.8	20.8–23.1 (21.6 ± 0.8)
Caudal peduncle depth	7.0–8.2 (7.4 ± 0.5)	6.4–10.4 (7.8 ± 1.2)	5.3	4.9–5.6 (5.1 ± 0.2)	7.4	5.1–7.4 (6.2 ± 0.8)
Pectoral‐fin length	12.9–19.0 (17.1 ± 1.6)	16.7–19.5 (18.1 ± 0.9)	17	15.6–17.7 (16.7 ± 0.8)	18.5	17.3–20.6 (18.9 ± 1)
Pectoral fin to pelvic fin	17.1–19.9 (18.6 ± 0.9)	16.5–21.7 (19.4 ± 1.7)	18.6	15.6–18.6 (17 ± 1.1)	17.1	14.4–17.1 (15.9 ± 0.7)
Body depth	20.8–23.5 (22.3 ± 0.8)	22.2–26.3 (23.6 ± 1.3)	21	19.7–24.5 (21.3 ± 1.6)	24.6	21.1–24.8 (23.4 ± 1.1)
Mid body depth	19.2–23.8 (21.5 ± 1.5)	18.8–26.1 (23 ± 1.8)	21.1	19.5–22.2 (21 ± 1)	21.9	18.6–23.2 (21.1 ± 1.3)
Head length	19.7–23.7 (22 ± 1.1)	22.8–27.6 (25.1 ± 1.6)	23.1	21.2–24.1 (22.4 ± 1)	24	20.4–24.0 (22.4 ± 1.3)
Length of the snout	38.3–42.5 (40.3 ± 1.3)	39.6–50.8 (44.2 ± 4.1)	39.6	39.5–42.3 (40.7 ± 1.1)	40.6	38.5–44.6 (41.4 ± 1.8)
Distance between nostrils on the same side	5.6–9.6 (6.8 ± 1.3)	5.2–10.0 (6.9 ± 1.4)	6.2	6.2–8.6 (7.2 ± 0.9)	5.9	4.6–5.9 (5.3 ± 0.4)
Orbit diameter	10.6–16.3 (14.2 ± 1.5)	9.0–14.2 (12 ± 1.7)	12.8	11.1–18.0 (15.6 ± 2.7)	14.9	10.0–19.7 (14.1 ± 2.4)
Head width	51.1–61.1 (55.8 ± 2.8)	44.2–62.6 (54.2 ± 6.8)	51.2	50.1–56.8 (52.6 ± 2.5)	54.5	50.4–58.2 (53.5 ± 2.3)
Distance between anterior nostrils	23.6–27.6 (25.1 ± 1.4)	16.5–31.1 (22.7 ± 4.7)	21.7	21.7–26.2 (23.3 ± 1.7)	24.8	20.5–27.4 (24.5 ± 2.1)
Interorbital width	35.5–43.7 (40.4 ± 2.5)	29.8–52.1 (39.5 ± 8.3)	39.1	38.1–43.8 (40.4 ± 2.1)	35.7	34.8–43.4 (38.5 ± 3)
Counts						
Number of scales around caudal peduncle	20	18 (14–22)	16	16	16	16 (12–16)
Number of scales along caudal peduncle	20 (19–24)	18 (17–22)	22	20 (19–24)	18	17 (17–20)
Number of scales along lateral line	79 (76–80)	70 (66–78)	84	78 (78–85)	75	74 (69–88)
Teeth on upper jaw	7 (6–7)	7 (6–7)	7	7 (6–8)	6	7 (6–7)
Teeth on lower jaw	8 (8–10)	8 (6–10)	9	10 (8–12)	3	6 (3–8)
Number of pectoral‐fin rays	10	10	10	10	10	10
Number of pelvic‐fin rays	6	6	6	6	6	6
X‐ray counts						
Number of specimens	8	5	_	7	_	12
First anal‐fin ray to first dorsal‐fin ray	3 (3–5)	3 (3–4)	5	5 (5–6)	5	6 (5–7)
Total vertebrae	43 (42–43)	40 (40–41)	46	47 (46–47)	43	43 (42–44)
Pre‐caudal vertebrae	19 (18–19)	18	21	20 (20–21)	19	19 (18–19)
Caudal vertebrae	24 (23–25)	22 (22–23)	25	27 (25–27)	24	24 (24–25)
Vertebrae before the first dorsal‐fin radial	20 (20–21)	19 (19–20)	22	23 (22–24)	21	20 (19–21)
Vertebrae before the first anal‐fin radial	19 (19–20)	18 (18–19)	21	21 (21–22)	19	19 (18–19)
Dorsal‐fin rays	19 (17–21)	19	23	23 (22–24)	23	24 (22–25)
Anal‐fin rays	24 (22–25)	22 (22–24)	30	28 (28–30)	27	27 (26–29)

*Note*: The ranges of the measurements are presented with the mean and standard deviation inside the parentheses. For the counts, the number next to the parentheses represents the mode, and the numbers in the parenthesis represent the range.

Rounded blunt snout, below eye level. Dorsal head profile convex from snout to back of head where it becomes gently inclined towards dorsal fin. Small sub‐terminal mouth in line with pectoral‐fin base. Small rounded chin swelling transitions into the concave ventral profile of head. Round orbit. Anterior and posterior nostrils laterally positioned, closer to snout tip than opercular opening, anterior to and arranged horizontally in line with orbit. Anterior nostril positioned slightly higher than posterior nostril. Small gill opening with soft skin cover adjacent to pectoral‐fin base. Bicuspid teeth: 6–7 in upper jaw and 6–9 in lower jaw.

Laterally compressed body with greatest width between gill covers. Fusiform body with greatest depth occurring between origin of dorsal and anal fins. Body tapers gently towards head, while tapering sharply from origins of both dorsal and anal fins; the thin caudal peduncle maintains same depth from its origin to roughly around half its length, then widens into two symmetrical lobes. Body covered with transparent membrane that becomes increasingly translucent to opaque towards head, dorsal and ventral surfaces in preserved specimens. Head without scales; but rest of the body is covered by small cycloid scales with reticulated striae. Lateral line originates above pectoral fin and forms a straight line to caudal peduncle. There are 60–74 scales along lateral line, 16–18 along caudal peduncle and 14–18 around caudal peduncle. Urogenital opening situated adjacent to origin of anal fin.

Rounded pectoral fin with 10 rays, extending just beyond origin of pelvic fin. Short pelvic fin with 6 rays. The first two dorsal and anal‐fin rays are unbranched, unsegmented and small. These two unbranched and unsegmented rays are followed by a single long unbranched and segmented ray, followed by numerous branched, segmented rays. The last dorsal‐ and anal‐fin ray is usually branched all the way to its base. Anal and dorsal fins set towards posterior of body. Anal‐fin origin is 6–9 fin rays anterior to dorsal‐fin origin. Dorsal‐fin rays: 18–21; anal‐fin rays: 23–26. In both dorsal and anal fins, anterior fin rays increase in length up to fourth and fifth rays; subsequent rays get progressively shorter. Caudal fin deeply forked with rounded lobes with bases covered in scales; distance from caudal‐fin flexion point to caudal‐fin tips is roughly equal to caudal peduncle length.

See Figure 10. Live colour: body colour ranges from brown to grey. Dorsal surface usually darker than the ventral surface. Dark vertical bar originates from origin of dorsal fin. Dark vertical bar originates from origin of dorsal fin. Dark blotch present near the flexion point of the caudal fin; dark vertical bar often visible just anterior to the caudal‐fin flexion point. Fins brown. Preserved in ethanol: body colour ranges from light to dark brown. Dorsal surface usually darker than the ventral surface. Dark vertical bar originates from origin of dorsal fin. Dark blotch presents near the flexion point of the caudal fin; dark vertical bar often visible just anterior to the caudal‐fin flexion point. Fins brown.

Determining the sex of adults can be done externally by examining their anal‐fin base. In males, the body wall is dorsally indented giving the dorsal margin of the anal fin a sigmoid curvature. In females, the body wall appears almost straight.

Total vertebrae: 40–42, pre‐caudal vertebrae: 18–19, caudal vertebrae: 22–24, vertebrae at first dorsal radial: 20–21, vertebrae at first anal radial: 18–19.

#### 4.2.5 Distribution

*H. pauciradiatus* was recorded from the mainstem channel of the Kwanza River and its north bank tributary, the Lucala River, in Angola (Figure 9).

### 4.3 *H. dolichorhynchus* sp. nov.

**FIGURE 11:**
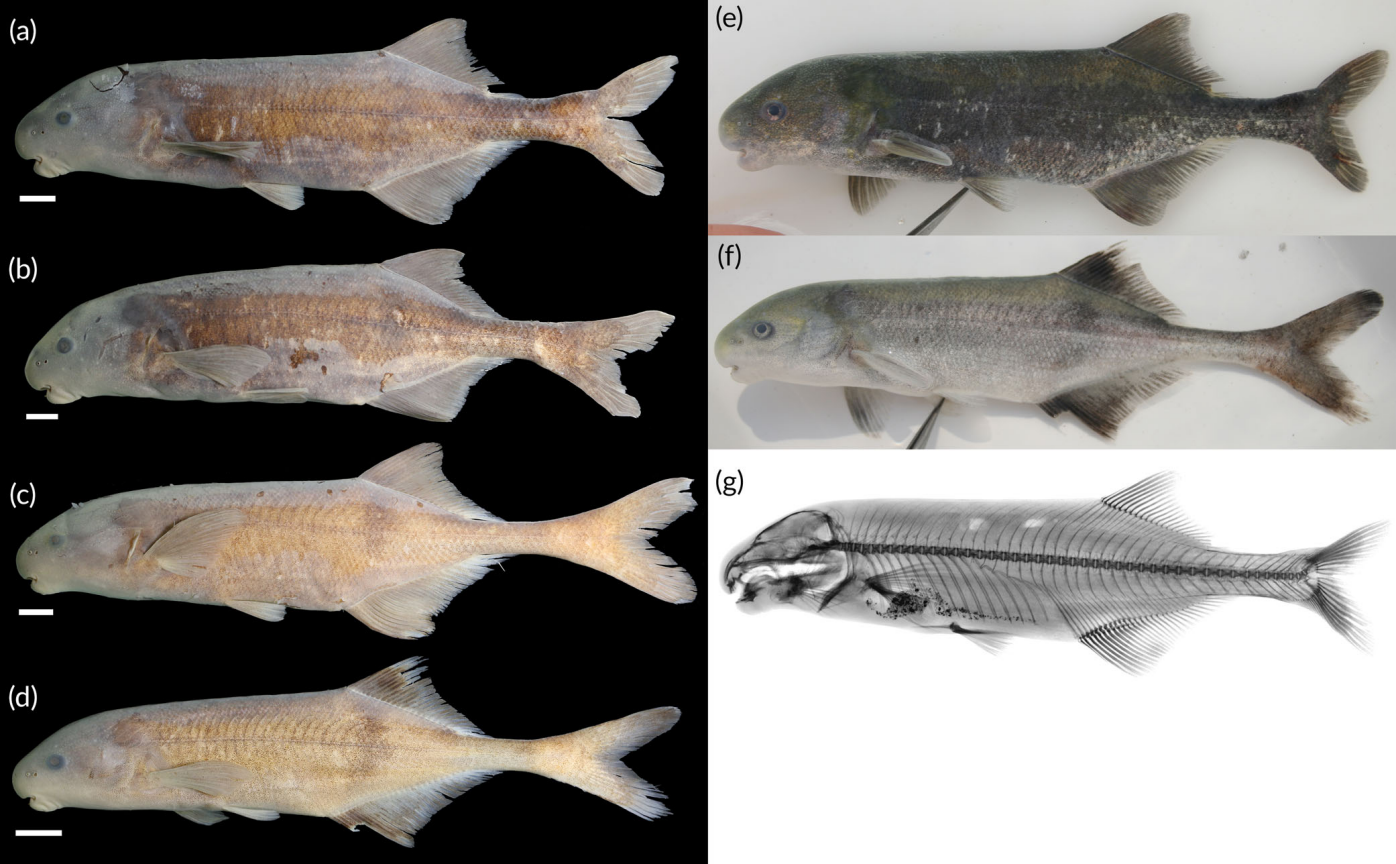
(a) Holotype of *Heteromormyrus dolichorhynchus* sp. nov. (SAIAB 85219), female 151.0 mm standard length (SL), from the Kwanza River; (b) paratype of *H. dolichorhynchus* sp. nov. (SAIAB 85219), male 155.0 mm SL, from the Kwanza River; (c) *H. dolichorhynchus* sp. nov. (SAIAB 84645), male 151.0 mm SL, from the Kwanza River; (d) *H. dolichorhynchus* sp. nov. (SAIAB 84645), female 117.0 mm SL, from the Kwanza River; (e, f) live pictures of *H. dolichorhynchus* sp. nov.; (g) X‐ray radiograph of holotype. Scale bar =1 cm.

https://zoobank.org/NomenclaturalActs/d6a799cc-01cf-4175-a062-60931370a6d5; Figure 11.

*Hippopotamyrus* sp. ‘K5’ in Mutizwa et al. (2021).

*Heteromormyrus* sp. ‘K5’ in Sullivan et al. (2022).

#### 4.3.1 Holotype

SAIAB 246301, 151.0 mm SL, farm below Lucala 2 bridge, 9° 25′ 30″ S, 14° 42′ 0″ E, Kwanza River system, Angola, E. Swartz and A. Chakona, 25 August 2008. BOLD genseq‐1 COI: SAFW620‐09.

#### 4.3.2 Paratypes

SAIAB 85219, 5 specimens 94.0–176.0 mm SL, farm below Lucala 2 bridge, 9° 25′ 30″ S, 14° 42′ 0″ E, Kwanza River system, Angola, E. Swartz and A. Chakona, 25 August 2008.

#### 4.3.3 Non‐type specimens

SAIAB 246348, 1 specimen 86.0 mm SL, Lucala River bridge, 9° 16′ 8″ S, 15° 14′ 49″ E, Kwanza River, Angola, E. Swartz and A. Chakona, 18 August 2008, BOLD genseq‐3 COI: SAFW492‐09, GenBank genseq‐3 cyt b: MW600879, S7 intron: MW756356, MW756357; SAIAB 84645, 5 specimens 73.1*–*150.9 mm SL, Terra Nova, 9° 46′ 44″ S, 14° 31′ 24″ E, Kwanza River system, Angola, E. Swartz, 9 October 2007, BOLD genseq‐3 COI: SAFW251‐08, GenBank genseq‐3 S7 intron: MW756354, MW756355.

#### 4.3.4 Diagnosis

*H. dolichorhynchus* sp. nov. has a unique elongate head which distinguishes it from its congeners. *H. dolichorhynchus* sp. nov. has nostrils aligned horizontally below the level of orbit readily distinguishing it from its congeners whose anterior nostrils are always positioned higher than the posterior nostrils, and both nostrils are horizontally in line with orbit. A higher number of dorsal‐fin rays further distinguish *H. dolichorhynchus* sp. nov. (22*–*25) from most of its congeners, which include *H. tangwenai* sp. nov. (17*–*20), *H. ndauorum* sp. nov. (19*–*20), *H. chilembwei* sp. nov. (18*–*20), *H. szaboi* (19), *H. xanekweorum* sp. nov. (18*–*20), *H. longilateralis* (17*–*21), *H. ansorgii* (18*–*21), *H. tavernei* (17–20) and *H. pauciradiatus* (18*–*21). A higher number of anal‐fin rays further distinguish *H. dolichorhynchus* sp. nov. (26*–*29) from *H. ndauorum* sp. nov. (23*–*24), *H. tangwenai* sp. nov. (20*–*25), *H. chilembwei* sp. nov. (23*–*25), *H. szaboi* (22*–*24), *H. xanekweorum* sp. nov. (22*–*24) and *H. longilateralis* (22*–*25). The number of anal‐fin rays between the leading ray and the one directly below the leading dorsal‐fin ray distinguishes *H. dolichorhynchus* sp. nov. (5–7) from *H. szaboi* (3–4) and *H. ndauorum* sp. nov. (3–4). The total vertebrae separate *H. dolichorhynchus* sp. nov. (42*–*44) from *H. angusticaudata* sp. nov. (46*–*47) and *H. szaboi* (40–41). A higher number of caudal vertebrae separate *H. dolichorhynchus* sp. nov. (24*–*25) from *H. szaboi* (22*–*23). A higher number of vertebrae before the first dorsal radial distinguish *H. dolichorhynchus* sp. nov. (18–19) from *H. chilembwei* sp. nov. (20). *H. dolichorhynchus* sp. nov. has 12–16 scales around the caudal peduncle, distinguishing it from *H. tavernei* (20–22), *H. longilateralis* (20) and *H. tangwenai* sp. nov. (18–20). *H. dolichorhynchus* sp. nov. (18.3%–21.7%TL) has a shallower body depth compared to *H. pappenheimi* (25.0%–27.3%TL). The lack of any clearly visible marks in the caudal peduncle further distinguishes *H. dolichorhynchus* sp. nov. from *H. ansorgii*, *H. pauciradiatus* and *H. angusticaudata* sp. nov. that have a clearly visible dark blotch present near the flexion point of the caudal fin and a dark vertical bar in the caudal peduncle; *H. longilateralis*, *H. szaboi*, *H. xanekweorum* sp. nov. and *H. tangwenai* sp. nov. have only a dark blotch present near the flexion point of the caudal fin.

#### 4.3.5 Description

Morphometric proportions and meristics are summarised in Table 3. Meristic counts of the holotype are provided in parentheses.

Rounded blunt snout, below eye level. Straight dorsal head profile forms obtuse angle with the gently inclined dorsal body profile. Small sub‐terminal mouth placed below the level of pectoral‐fin base. Small rounded chin swelling transitions into deep concave ventral profile of head. Round orbit. Anterior and posterior nostrils laterally positioned, closer to snout tip than opercular opening, anterior to and arranged horizontally below orbit. Small gill opening with soft skin cover adjacent to pectoral‐fin base. Bicuspid teeth: 6*–*7 (6) in upper jaw and 3*–*8 (3) in lower jaw.

Laterally compressed body with greatest breadth occurring across gill covers. Fusiform body with greatest depth occurring between origin of dorsal and anal fins. Body tapers gently towards head, while tapering sharply from origins of both dorsal and anal fins to produce a relatively thin caudal peduncle. Caudal peduncle maintains same thin depth from its origin to roughly around half its length, then it gradually widens into two symmetrical lobes. Body covered with transparent membrane that becomes increasingly translucent to opaque towards head, dorsal and ventral surfaces in preserved specimens. Head without scales; but the rest of the body is covered with small cycloid scales with reticulated striae. Lateral line originates approximately above pectoral fin and forms a straight line to caudal peduncle. There are 69*–*88 (75) scales along lateral line, 17*–*20 (18) along caudal peduncle and 12*–*16 (16) around caudal peduncle. Urogenital opening situated adjacent to origin of anal fin.

Rounded pectoral fin with 10 rays, extending just beyond origin of pelvic fin. Short pelvic fin with 6 rays. The first two dorsal and anal‐fin rays are unbranched, unsegmented and small. These two unbranched and unsegmented rays are followed by a single long unbranched and segmented ray, followed by numerous branched, segmented rays. The last dorsal‐ and anal‐fin ray is usually branched all the way to its base. Anal and dorsal fins set towards posterior of body. Anal‐fin origin is 5–7 (5) fin rays anterior to dorsal‐fin origin. Dorsal‐fin rays: 22*–*25 (23); anal‐fin rays: 26*–*29 (27). In both dorsal and anal fins, anterior fin rays increase in length up to fourth and fifth rays; subsequent rays get progressively shorter. Caudal fin deeply forked with rounded lobes with bases covered in scales; distance from caudal‐fin flexion point to caudal‐fin tips is roughly equal to caudal peduncle length.

See Figure 11. Live colour: body colour ranges from silver to grey. Dorsal surface usually darker than the ventral surface. Dark vertical bar originates from origin of dorsal fin. Dark vertical bar originates from origin of dorsal fin, barely visible in darker specimens. Thin curved vertical bars on anterior portion of flank most conspicuous in slivery specimens. Fins grey, darker than the body in silvery specimens. Preserved in ethanol: body colour ranges from light to dark brown. Dorsal surface usually darker than ventral surface. Dark vertical bar originates from origin of dorsal fin, barely visible in darker specimens. Thin curved vertical bars conspicuous in the anterior portion of flank. No clearly visible marking in the caudal peduncle.

Determining the sex of adults can be done externally by examining their anal‐fin base. In males, the body wall is dorsally indented giving the dorsal margin of the anal fin a sigmoid curvature. In females, the body wall appears almost straight.

Total vertebrae: 42*–*44 (43), pre‐caudal vertebrae: 18*–*19 (18), caudal vertebrae: 24–25 (25), vertebrae at first dorsal radial: 19–21(19), vertebrae at first anal radial: 18*–*19 (18).

#### 4.3.6 Distribution

*H. dolichorhynchus* was recorded from the mid‐reaches of the Kwanza River and its north bank tributary, the Lucala River, Angola (Figure 9).

#### 4.3.7 Etymology

The name is the combination of the Greek words *dolicho*, meaning elongated or long, and *rhynchos*, meaning beak or snout. This is a reference to the unique elongate head of this species, which distinguishes it from its congeners.

#### 4.3.8 Remarks

*H. dolichorhynchus* has two morphs, one that has a deeper caudal peduncle with 16 scales around the caudal peduncle (Figure 11a,b), and the other has a narrower caudal peduncle with 12 scales (Figure 11c,d). These two morphs are genetically and morphologically similar, except for the caudal peduncle depth and caudal scale counts. The specimens (SAIAB 85219, SAIAB 246348) with the deeper caudal peduncle and higher number of scales were all collected from the Lucala River, a north bank tributary of the Kwanza River. The specimens (SAIAB 84645) with the narrower caudal peduncle and fewer caudal scales were collected from the mainstem Kwanza River. There are no obvious natural barriers between the collection sites of the different morphs. However, the Cambambe Hydroelectric Power Station, a dam on the Kwanza River mainstem completed in 1963, currently separates these morphs. Intraspecific differences in caudal peduncle depth have been linked to differences in water conductivity. However, water quality variables were not recorded during the 2007 and 2008 surveys. This will need to be ascertained in future surveys.

### 4.4 *H. angusticaudata* sp. nov

**FIGURE 12:**
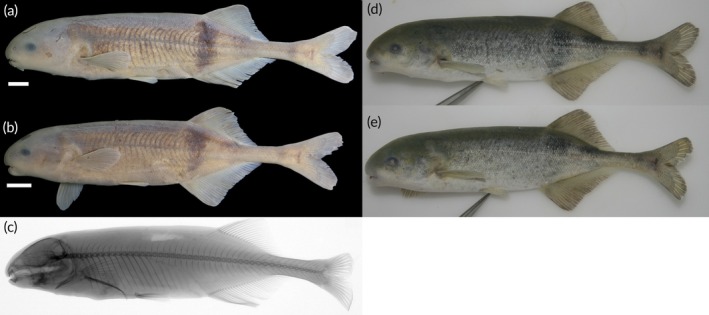
(a) Holotype of *Heteromormyrus angusticaudata* sp. nov. (SAIAB 85039), 152.5 mm standard length (SL), from the Kwanza River; (b) paratype of *H. angusticaudata* sp. nov. (SAIAB 85039), 149.0 mm SL, from the Kwanza River, southern Africa; (c) X‐ray radiograph of holotype; (d, e) live pictures of *H. angusticaudata* sp. nov. Scale bar =1 cm.

https://zoobank.org/NomenclaturalActs/43fa6d0f-461b-4933-9ad5-be3a36e10b58; Figure 12.

*Hippopotamyrus* sp. ‘K1’ in Mutizwa et al. (2021).

*Heteromormyrus* sp. ‘K1’ in Sullivan et al. (2022).

#### 4.4.1 Holotype

SAIAB 246302, 118.3 mm SL, northern track between Tchuimbo and Kutato, 12° 16′ 38″ S, 16° 21′ 3″ E, Angola, E. Swartz and A. Chakona, 12 August 2008; BOLD genseq‐1 COI: SAFW399‐08; GenBank genseq‐1 S7 intron: MW756324.

#### 4.4.2 Paratypes

SAIAB 85039, 6 specimens 94.8*–*152.5 mm SL, northern track between Tchuimbo and Kutato, 12° 16′ 38″ S, 16° 21′ 3″ E, Kwanza River system, Angola, E. Swartz and A. Chakona, 2 August 2008, BOLD genseq‐2 COI: SAFW398‐08, GenBank genseq‐2 S7 intron: MW756325.

#### 4.4.3 Non‐type specimens

SAIAB 85147, 1 specimen 114.8 mm SL, below Calema Falls, 09° 53′ 24″ S, 16° 18′ 16″ E, Angola, E. Swartz and A. Chakona, 21 August 2008; BOLD genseq‐3 COI: SAFW452‐08. SAIAB 85169, 1 specimen 29.6 mm SL, just above Calema Falls 09° 53′ 28″ S,16° 18′ 26″ E, Angola, E. Swartz and A. Chakona, 22 August 2008, BOLD genseq‐3 COI: SAFW545‐08, GenBank genseq‐3 S7 intron: MW756322, MW756323.

#### 4.4.4 Diagnosis

*H. angusticaudata* sp. nov. possesses the highest number of vertebrae (46*–*47), a character that differentiates this species from all its congeners in southern Africa: *H. tangwenai* sp. nov. (41*–*42), *H. ndauorum* sp. nov. (42), *H. chilembwei* sp. nov. (43), *H. szaboi* (40*–*41), *H. xanekweorum* sp. nov. (41*–*42), *H. longilateralis* (42*–*43), *H. ansorgii* (42*–*44), *H. dolichorhynchus* (42*–*44), *H. pappenheimi* (42*–*43) and *H. pauciradiatus* (40*–*42). The higher number of anal‐fin rays further distinguishes *H. angusticaudata* sp. nov. (28*–*30) from *H. ndauorum* sp. nov. (23*–*24), *H. tavernei* (22–23), *H. tangwenai* sp. nov. (20*–*25), *H. chilembwei* sp. nov. (23*–*25), *H. szaboi* (22*–*24), *H. xanekweorum* sp. nov. (22*–*24), *H. longilateralis* (22*–*25), *H. ansorgii* (24*–*27) and *H. pauciradiatus* (23*–*26). The number of anal‐fin rays between the leading ray and the one directly below the leading dorsal‐fin ray in *H. angusticaudata* sp. nov. (5–6) further distinguishes this species from *H. szaboi* (3–4) and *H. ndauorum* sp. nov. (3–4). A higher number of dorsal‐fin rays separate *H. angusticaudata* sp. nov. (22–24) from *H. tavernei* (17–20), *H. longilateralis* (17–21), *H. szaboi* (19), *H. ansorgii* (18–21), *H. xanekweorum* sp. nov. (18–20), *H. ndauorum* sp. nov. (18–20), *H. tangwenai* sp. nov. (17–20) and *H. chilembwei* sp. nov. (18–20). A lower number of scales around the caudal peduncle separate *H. angusticaudata* sp. nov. (16) from *H. tavernei* (20–22), *H. tangwenai* sp. nov. (18–20) and *H. longilateralis* (20). A narrow caudal peduncle distinguishes *H. angusticaudata* sp. nov. (4.9%*–*5.6%SL) from *H. pauciradiatus* (7.3%–9.2%SL), *H. longilateralis* (7.0%–8.2%SL), *H. szaboi* (6.4%–10.4%SL), *H. ansorgii* (6.0%–7.3%SL), *H. chilembwei* sp. nov. (5.9%–6.2%SL), *H. tangwenai* sp. nov. (6.3%–7.9%SL) and *H. ndauorum* sp. nov. (6.7%–7.5%SL). *H. angusticaudata* sp. nov. is readily distinguished from its congeners except *H. dolichorhynchus*, *H. longilateralis* and *H. xanekweorum* sp. nov. by a series of thin curved vertical bars that are more conspicuous in the anterior portion of the flank. The combination of a dark blotch present near the flexion point of the caudal fin and a dark vertical bar in the caudal peduncle further distinguishes *H. angusticaudata* sp. nov. from *H. dolichorhynchus*, *H. chilembwei* sp. nov. and *H. ndauorum* sp. nov. that do not have any clearly visible marks on the caudal peduncle; *H. longilateralis*, *H. szaboi*, *H. tangwenai* sp. nov. and *H. xanekweorum* sp. nov. have only a dark blotch present near the flexion point of the caudal fin.

#### 4.4.5 Description

Proportional measurements and meristics are summarised in Table 4. Holotype meristic counts are presented in parentheses.

Blunt snout, below eye level that gradually blends into convex dorsal profile of body. Small sub‐terminal mouth occurring below the level of pectoral‐fin base. Small rounded chin swelling transitions into concave ventral profile of head. Round orbit. Anterior and posterior nostrils laterally positioned, closer to snout tip than opercular opening, anterior to and arranged horizontally in line with orbit. Anterior nostril positioned slightly higher than posterior nostril. Small gill opening with soft skin cover adjacent to the pectoral‐fin base. Bicuspid teeth: 6*–*7 (7) in upper jaw and 8*–*12 (9) in lower jaw.

Laterally compressed body with greatest breadth occurring across gill covers. Fusiform body with greatest depth occurring between origin of dorsal and anal fins. Body tapers gently towards head, while tapering sharply from origins of both dorsal and anal fins to produce relatively thin caudal peduncle. Caudal peduncle maintains same thin depth from origin to roughly around half its length, gradually widens into two symmetrical lobes. Body covered with transparent membrane that becomes increasingly translucent to opaque towards head, dorsal and ventral surfaces in preserved specimens. Head without scales; the rest of the body is covered with small cycloid scales with reticulated striae. Lateral line originates approximately above pectoral fin and forms a straight line to the caudal peduncle. There are 78*–*85 (84) scales along the lateral line, 19*–*24 (22) of them along caudal peduncle and 16 around caudal peduncle. Urogenital opening situated adjacent to origin of anal fin.

Rounded pectoral fin with 10 rays, extending just beyond pelvic‐fin origin. Short pelvic fin with 6 rays. The first two dorsal‐ and anal‐fin rays are unbranched, unsegmented and small. These two unbranched and unsegmented rays are followed by a single long unbranched and segmented ray, followed by numerous branched, segmented rays. The last dorsal‐ and anal‐fin ray is usually branched all the way to its base. Anal and dorsal fins set towards the posterior of the body. Anal‐fin origin is 5–6 (5) fin rays anterior to the dorsal‐fin origin. Dorsal‐fin rays: 22*–*24 (23); anal‐fin rays: 28*–*30 (30). In both dorsal and anal fins, the anterior fin rays increase in length up to fourth and fifth rays, then subsequent rays get progressively shorter. Caudal fin deeply forked with rounded lobes with bases covered in scales; distance from caudal‐fin flexion point to caudal‐fin tips is roughly equal to caudal peduncle length.

See Figure 12. Live colour: body colour ranges from silver to grey. Dorsal surface usually darker than the ventral surface. A series of thin curved vertical bars more conspicuous in the anterior portion of flank. Dark vertical bar originates from origin of dorsal fin. Dark blotch presents near the flexion point of the caudal fin and a dark vertical bar just anterior to this blotch. Fins grey, a shade lighter than the body. Preserved in ethanol: body colour light brown. Dorsal surface usually darker than ventral surface. Dark vertical bar originates from origin of dorsal fin. Dark blotch presents near the flexion point of the caudal fin and a dark vertical bar just anterior to this blotch. A series of clearly visible thin curved vertical bars more conspicuous in the anterior portion of flank, lateral line similarly darkened as well. Lightly coloured fins.

Total vertebrae: 46*–*47 (46), pre‐caudal vertebrae: 20–21 (20), caudal vertebrae: 26*–*27 (27), vertebrae at first dorsal radial: 22*–*24 (22), vertebrae at first anal radial: 21*–*22 (21).

Determining the sex of adults can be done externally by examining their anal‐fin base. In males, the body wall is dorsally indented, giving the dorsal margin of the anal fin sigmoid curvature. In females, the body wall appears almost straight. All the specimens of *H. angusticaudata* examined in this study had a straight body wall at their anal‐fin base. Because no verification of the sex of the specimens was carried out (e.g., by dissection of gonads), it was not possible to confirm whether this species is sexually dimorphic. It is possible that there were no sexually mature males among the examined specimens.

#### 4.4.6 Distribution

The species was collected in the upper and middle sections of the Kwanza River mainstem; see Figure 9.

#### 4.4.7 Etymology

The species name means narrow‐tailed, and it is a compound word composed of the Latin words *angustus*, meaning narrow, and *cauda*, meaning tail. This is a reference to the very thin caudal peduncle of this species.

### 4.5 *H. xanekweorum* sp. nov

**FIGURE 13:**
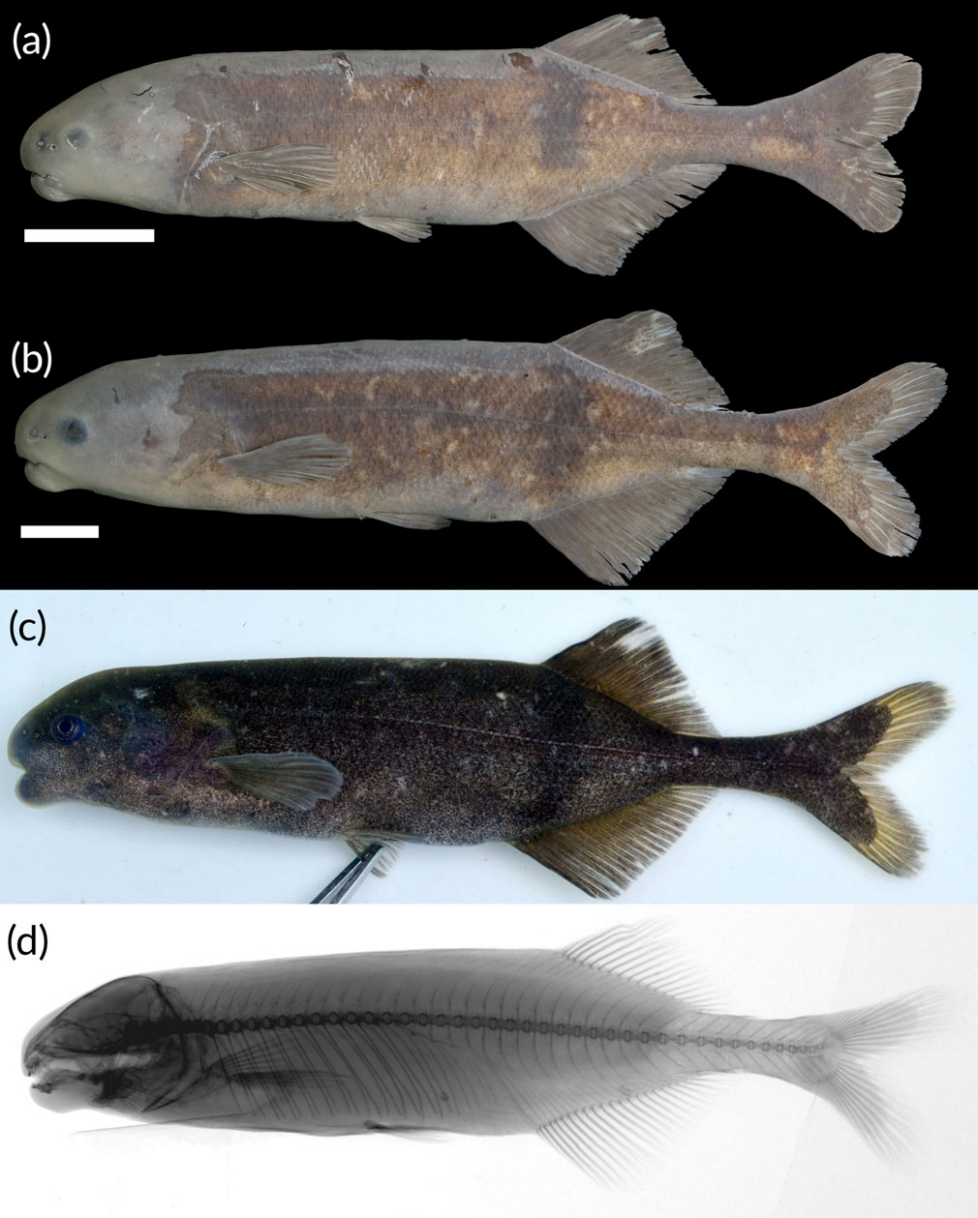
(a) Holotype of *Heteromormyrus xanekweorum* sp. nov. (SAIAB 186792), male 66.7 mm standard length (SL), from the Okavango River, southern Africa; (b) paratype of *H. xanekweorum* sp. nov. (SAIAB 186792), female 99 mm SL, from the Okavango River, southern Africa; (c) live picture of *H. xanekweorum* sp. nov.; (d) X‐ray radiograph of holotype. Scale bar =1 cm.

https://zoobank.org/NomenclaturalActs/e59930ae-52a7-4adf-9fee-6a6bd16d18dc; Figure 13.

*Hippopotamyrus* sp. ‘OK’ in Mutizwa et al. (2021).

*Heteromormyrus* sp. ‘OK’ in Sullivan et al. (2022).

#### 4.5.1 Holotype

SAIAB 246300, 84.5 mm SL, just south of Chitembo, Chitembo River, 13° 31′ 41.1″ S, 16° 45′ 31.4″ E, Okavango River system, Angola, R. Bills, P. Skelton, F. de Almeida and M. Domingos, 16 May 2012, BOLD genseq‐1 COI: ANGFW139‐12.

#### 4.5.2 Paratypes

SAIAB 186792, 9 specimens 37.0*–*118.1 mm SL, just south of Chitembo, Chitembo River, 13° 31′ 41.1″ S, 16° 45′ 31.4″ E, Okavango River system, Angola, R. Bills, P. Skelton, F. de Almeida and M. Domingos, 16 May 2012, BOLD and GenBank genseq‐2 COI: ANGFW138‐12, MW600872, MW600873, GenBank genseq‐2 cyt *b*: MW600903, S7 intron: MW756320, MW756321.

#### 4.5.3 Non‐type specimens

SAIAB 186693, 4 specimens 32.0–113.0 mm SL, bridge of 1000 Mines north of Mambue, ‘Bie’, Cacuchi River, 13° 35′ 39.6″ S, 16° 52′ 49.8″ E, Okavango River, Angola, R. Bills, P. Skelton, F. de Almeida and M. Domingos, 11 May 2012, BOLD genseq‐3 COI: ANGFW053‐12; SAIAB 186894, 1 specimen 38.0 mm SL, Cuanavale River just above confluence, 15° 08′ 08.5″ S, 19° 11′ 44.6″ E, Okavango River, Angola, R. Bills, P. Skelton, F. de Almeida and M. Domingos, 19 May 2012, GenBank genseq‐3 COI: MW600877, cyt *b*: MW600898; SAIAB 186651, 3 specimens 39–41 mm SL, Square Ponglesia‐Cubango, Quebe River, 14° 56′ 22.9″ S, 17° 43′ 07.7″ E, Okavango River system, Angola, R. Bills, P. Skelton, F. de Almeida, 9 May 2012, BOLD genseq‐3 COI: ANGFW019‐12, GenBank genseq‐3 cyt *b*: MW600899; SAIAB 202504, 1 specimen 84.0 mm SL, fish eagle camp, Cuito River, 13° 46′ 17.00″ S, 18° 34′ 48.00″ E, Okavango River system, Angola, A. Costa, 20 June 2015, GenBank genseq‐3 COI: MW600876; SAIAB 204769, 1 specimens 72.0 mm SL, Rapid 1, Cubango River, 13° 02′ 41.12″ S, 16° 22′ 29.93″ E, Okavango River system, Angola, P. Skelton and B. van der Waal, 8 May 2017, GenBank genseq‐3 COI: MW600870, cyt *b*: MW600893; SAIAB 203980, 1 specimen 51.0 mm SL, below bridge on Cuchi River, 14° 42′ 09.8″ S, 17° 22′ 43.1″ E, Okavango River system, Angola, B. van de Waal and N. Mazungula, 15 October 2016, GenBank genseq‐3 COI: MW600869; SAIAB 187022, 1 specimen 39.0 mm SL, Popa, Okavango River, 18° 07′ 18.2″ S, 21° 35′ 00.2″ E, Okavango River system, Namibia, R. Bills; P. Skelton, 24 May 2012, BOLD genseq‐3 COI: ANGFW227‐12, GenBank genseq‐3 cyt *b*: MW600900; SAIAB 204843, 3 specimens 48.0–61.0 mm SL, Kavango mission rapids camp, Cubango River, 13° 19′ 40.14″ S, 16° 24′ 39.82″ E, Okavango River system, Angola, P Skelton and B van der Waal on 11 May 2017, GenBank genseq‐3 COI: MW600871, cyt *b*: MW600897; SAIAB 186726, 3 specimens 28.0–92.0 mm SL, Cacuchi tributary (culvet), Mumbue, ‘Bie’, Watana River, 13° 16′ 46.7″ S, 16° 44′ 46.1″ E, Okavango River system, Angola, R. Bills; P. H. Skelton; M. Domingos; F. de Almeida on 13 May 2012, BOLD genseq‐3 COI: ANGFW090‐12, GenBank genseq‐3 cyt *b*: MW600894; SAIAB 202483, 1 specimen 66.0 mm SL, Hippo camp, 13° 08′ 29.00″ S, 18° 29′ 08.00″ E, Okavango River system, Angola, A. Costa on 11 June 2015, GenBank genseq‐3 COI: MW600874, cyt *b*: MW600895, S7 intron: MW756362, MW756363; SAIAB 203161, 1 specimen 57.0 mm SL, Kalva and Cuito confluence, Cuito River, 12.74878° S, 18.354334° E, Okavango River system, Angola, A. Costa on 25 Feb 2016, GenBank genseq‐3 COI: MW600875, cyt *b*: MW600896.

#### 4.5.4 Diagnosis

The number of anal‐fin rays between the leading ray and the one directly below the leading dorsal‐fin ray distinguishes *H. xanekweorum* sp. nov. (4–5) from *H. pappenheimi* (6–9), *H. pauciradiatus* (6–9), *H. ansorgii* (6–9), *H. tangwenai* sp. nov. (6–7) and *H. chilembwei* sp. nov. (6–7). A lower number of anal‐fin rays further separate *H. xanekweorum* sp. nov. (22*–*24) from *H. angusticaudata* (28*–*30), *H. dolichorhynchus* (26*–*29) and *H. pappenheimi* (26*–*28). A higher number of scales around caudal peduncle separate *H. xanekweorum* sp. nov. (16–20) from *H. pappenheimi* (12*–*14). A lower number of caudal vertebrae differentiate *H. xanekweorum* sp. nov. (22*–*24) from *H. angusticaudata* (26*–*27). The number of vertebrae before first dorsal radial further distinguishes *H. xanekweorum* sp. nov. (20*–*21) from *H. angusticaudata* (22*–*24). The vertebrae before first anal radial further separate *H. xanekweorum* sp. nov. (19*–*20) from *H. pappenheimi* (18). *H. xanekweorum* sp. nov. possesses a narrower caudal peduncle depth (5.0%–6.8%SL) that distinguishes it from *H. longilateralis* (7.0%–8.2%SL), *H. tavernei* (6.9%–8.6%SL) and *H. pauciradiatus* (7.3%–9.2%SL). *H. xanekweorum* sp. nov. (19.8%*–*22.8%SL) has a narrower body depth compared to *H. pauciradiatus* (23.1%–27.9%SL) and *H. szaboi* (22.4%–26.3%SL). A shallower caudal peduncle depth further separates *H. xanekweorum* sp. nov. (5.0%–6.8%SL) from *H. szaboi* (6.4%–10.4%SL). A dark blotch near flexion point of the caudal fin distinguishes *H. xanekweorum* sp. nov. from *H. dolichorhynchus*, *H. chilembwei* sp. nov. and *H. ndauorum* sp. nov. that do not have any clearly visible marks on caudal peduncle; *H. ansorgii*, *H. pauciradiatus* and *H. angusticaudata* have only a clearly visible dark blotch present near flexion point of the caudal fin and a dark vertical bar on caudal peduncle.

#### 4.5.5 Description

Proportional measurements and meristics are summarised in Table 5. Holotype meristic counts are presented in parentheses.

**TABLE 5:** Proportional measurements and meristic data for *Heteromormyrus* examined in this study.

	*Heteromormyrus xanekweorum* sp. nov. holotype	*H. xanekweorum* sp. nov.	*Heteromormyrus chilembwei* sp. nov. holotype	*H. chilembwei* sp. nov.	*Heteromormyrus tangwenai* sp. nov. holotype	*H. tangwenai* sp. nov.	*Heteromormyrus ndauorum* sp. nov. holotype	*H. ndauorum* sp. nov.
Number of specimens	–	23	–	7	–	19	–	7
Total length	94.9	41.8–130.3 (68.5 ± 23.5)	71.7	38.3–76.0 (58.5 ± 13.7)	114.8	57.3–114.8 (81 ± 16.5)	166	34.7–166 (103.3 ± 63.2)
Standard length	84.5	37.0–118.1 (61.5 ± 21)	65.1	33.9–69.2 (52.6 ± 12.6)	102.2	49.2–102.2 (72.1 ± 14.8)	149.2	30.7–149.2 (92.4 ± 56.9)
Head length	20.4	9.3–25.7 (14.2 ± 4.3)	14.9	8.4–14.9 (12.1 ± 2.5)	23.6	11.7–23.6 (16.7 ± 3.6)	31.7	7.6–32.2 (20.5 ± 11.8)
Pre‐dorsal length	64.5	62.7–66.7 (64.4 ± 0.7)	64.5	63.2–67.0 (64.9 ± 1.5)	65.3	62.7–67.8 (65.3 ± 1.3)	64.6	62.2–66.3 (64.3 ± 1.5)
Pre‐anal length	61.4	58.1–65.3 (60.7 ± 1.6)	59	57.6–62.8 (59.7 ± 1.6)	60.7	59.0–62.2 (60.9 ± 1.1)	60.5	58.1–64.0 (61.1 ± 2.2)
Pre‐pelvic length	42.6	39.8–44.6 (42.3 ± 1.2)	41.3	40.3–44.1 (41.4 ± 1.3)	41	40.0–44.0 (42 ± 0.9)	41.1	38.6–46.1 (42.4 ± 2.6)
Pre‐pectoral length	24.9	23.5–28.8 (26.3 ± 1.5)	23.4	23.4–29.1 (26.8 ± 2)	24.6	22.2–28.3 (26.1 ± 1.7)	22.6	22.5–30.5 (25.8 ± 3.4)
Pectoral fin to anal fin	36.6	20.1–41.7 (35.8 ± 1.8)	36.4	33.2–36.4 (34.9 ± 1.4)	37.8	33.6–38.2 (36 ± 1.3)	39.2	31.9–40.8 (36.5 ± 3.5)
Pelvic fin to anal fin	18.7	16.5–22.2 (18.4 ± 1.2)	18	18.0–18.6 (18.2 ± 0.2)	20.5	17.3–20.9 (18.9 ± 0.9)	20.4	16.4–21.1 (19.3 ± 1.7)
Pectoral fin to dorsal fin	40.9	39.0–45.3 (41.2 ± 1.2)	41.8	38.2–42.7 (40.7 ± 1.7)	43.9	39.2–43.9 (41.6 ± 1.3)	44.3	38.8–45.0 (41.9 ± 2.5)
Dorsal fin to pelvic fin	29.9	29.2–32.8 (30.8 ± 1)	31.1	29.3–31.1 (30.3 ± 0.8)	33.4	30.1–33.4 (31.8 ± 1.1)	34.4	30.3–34.4 (32.3 ± 1.6)
Dorsal‐fin base length	18.2	14.4–21.4 (17 ± 1.5)	15	13.2–15.1 (14.5 ± 0.7)	15.6	13.6–17.0 (15.7 ± 0.9)	18.5	15.2–18.5 (17.1 ± 1.2)
Anal‐fin base length	21.3	16.1–22.2 (19.6 ± 1.4)	18.5	16.6–19.9 (18.5 ± 1)	20.5	17.4–21.1 (19.1 ± 1)	21	17.3–21.9 (20.4 ± 1.6)
Dorsal fin to caudal fin	39.5	33.0–40.3 (37.4 ± 1.5)	37.9	35.7–39.3 (37.2 ± 1.2)	37.3	36.1–39.9 (37.8 ± 1.1)	38.6	36.0–41.6 (39 ± 1.5)
Caudal peduncle length	19.5	18.1–23.4 (20.9 ± 1.3)	21.5	21.5–25.3 (23.4 ± 1.5)	21.9	19.8–23.7 (22.2 ± 1.1)	19.1	19.1–22.1 (20.9 ± 1.4)
Caudal peduncle depth	5.2	5.0–6.8 (6.1 ± 0.6)	6.2	5.9–6.2 (6.1 ± 0.1)	7	6.3–7.9 (7.1 ± 0.4)	7.1	6.7–7.5 (7.1 ± 0.3)
Pectoral‐fin length	17.7	15.0–21.7 (17.9 ± 1.5)	17.1	16.2–18.4 (17.6 ± 0.8)	16	15.2–21.3 (17.6 ± 1.6)	19.7	17.4–21.0 (19.3 ± 1.3)
Pectoral fin to pelvic fin	18.9	16.0–20.8 (17.9 ± 1.1)	18.2	14.4–18.9 (17.3 ± 1.5)	17.7	16.1–19.0 (17.8 ± 0.7)	20.9	16.0–21.8 (18.6 ± 2.4)
Body depth	22.6	19.8–22.8 (21.8 ± 0.8)	21.3	20.4–21.8 (20.9 ± 0.5)	23	21.0–23.9 (22.7 ± 0.8)	21.6	21.6–24.3 (23 ± 1)
Mid body depth	22	16.8–22.2 (20.6 ± 1.2)	22.6	18.7–22.6 (20.3 ± 1.3)	22.8	20.8–24.6 (22.6 ± 0.9)	24.3	20.0–24.3 (22.1 ± 1.6)
Head length	24.2	20.8–26.0 (23.4 ± 1.5)	22.9	21.5–24.7 (23.2 ± 1.1)	23	21.6–24.9 (23.1 ± 1)	21.3	21.3–25.4 (22.9 ± 1.6)
Length of the snout	38.7	33.4–46.7 (40.9 ± 3.1)	45.5	42.6–50.2 (46.5 ± 2.5)	43.2	41.1–53.5 (46.9 ± 3.9)	45.9	38.0–51.0 (42.5 ± 4.6)
Distance between nostrils on the same side	5.4	5.4–10.2 (7.7 ± 1.2)	9.5	7.5–12.7 (9.6 ± 1.6)	7.9	7.5–11.8 (8.9 ± 1.2)	6.7	6.7–11.1 (8 ± 1.6)
Orbit diameter	9.1	8.2–15.8 (11.6 ± 1.9)	13.1	8.6–13.1 (11.3 ± 1.7)	13.3	10.5–18.6 (13.8 ± 2.2)	11.1	10.9–15.0 (12.6 ± 1.5)
Head width	50.7	50.7–64.7 (56.5 ± 3.3)	60.3	55.3–63.1 (59.9 ± 2.6)	61.1	55.4–65.0 (59.8 ± 2.9)	60.7	54.5–67.2 (61.1 ± 4.5)
Distance between anterior nostrils	19.6	19.6–30.2 (26 ± 3.1)	29.9	26.3–29.9 (28.6 ± 1.3)	28.3	24.3–31.8 (28.4 ± 2.1)	27.8	24.2–29.7 (26.9 ± 1.9)
Interorbital width	33.6	33.6–50.0 (43.7 ± 4.2)	51.6	46.4–51.6 (49.3 ± 1.6)	48.9	41.7–54.4 (47.9 ± 3)	46	41.9–52.0 (45 ± 3.8)
Counts								
Number of scales around caudal peduncle	20	20 (16–20)	16	16	20	20 (18–20)	16	16
Number of scales along caudal peduncle	20	20 (16–23)	22	22 (21–23)	21	21 (18–22)	16	18 (16–20)
Number of scales along lateral line	76	78 (64–86)	82	82 (82–84)	83	71 (71–83)	78	72 (72–78)
Teeth on upper jaw	7	7 (6–7)	7	7 (7–8)	7	7 (4–8)	7	7 (5–9)
Teeth on lower jaw	8	8 (6–10)	9	8 (8–9)	8	8 (4–10)	8	8 (8–9)
Number of pectoral‐fin rays	10	10	10	10	10	10	10	10 (9–10)
Number of pelvic‐fin rays	6	6	6	6	6	6	6	6
X‐ray counts								
Number of specimens	–	7	–	3	–	14	–	3
First anal‐fin ray to first dorsal‐fin ray	5	5 (4–5)	7	7 (6–7)	6	6 (6–7)	4	4 (3–4)
Total vertebrae	41	41 (41–42)	43	43	41	41 (41–42)	42	42
Pre‐caudal vertebrae	19	19 (18–19)	20	19 (18–20)	18	18 (17–19)	18	18 (17–18)
Caudal vertebrae	22	22 (22–24)	23	24 (24–25)	23	23 (22–25)	24	24 (24–25)
Vertebrae before the first dorsal‐fin radial	21	20 (20–21)	21	22 (21–22)	20	20 (19–21)	20	20 (19–20)
Vertebrae before the first anal‐fin radial	19	19 (19–20)	20	19 (18–20)	18	19 (18–20)	18	19 (18–19)
Dorsal‐fin rays	18	19 (18–20)	20	20 (18–20)	18	19 (17–20)	20	20 (19–20)
Anal‐fin rays	22	23 (22–24)	25	23 (23–25)	22	24 (20–25)	23	23 (23–24)

*Note*: The ranges of the measurements are presented with the mean and standard deviation inside the parentheses. For the counts, the number next to the parentheses represents the mode, and the numbers in the parenthesis represent the range.

Rounded blunt snout, below eye level. Straight dorsal head profile forms obtuse angle with the gently inclined dorsal body profile. Small sub‐terminal mouth occurring below the level of pectoral‐fin base. Small rounded chin swelling transitions into deep concave ventral profile of head. Round orbit. Anterior and posterior nostrils laterally positioned, closer to snout tip than opercular opening, anterior to and arranged horizontally in line with orbit. Anterior nostril positioned slightly higher than posterior nostril. Small gill opening with soft skin cover adjacent to pectoral‐fin base. Bicuspid teeth: 6*–*7 (7) in upper jaw and 6*–*10 (8) in lower jaw.

Laterally compressed body with greatest breadth occurring across gill covers. Fusiform body with greatest depth occurring between origin of dorsal and anal fins. Body tapers gently towards head, while tapering sharply from origins of both dorsal and anal fins to produce a relatively thin caudal peduncle. Caudal peduncle maintains same thin depth from its origin to roughly around half its length, then it gradually widens into two symmetrical lobes. Body covered with transparent membrane that becomes increasingly translucent to opaque towards head, dorsal and ventral surfaces in preserved specimens. Head without scales, the rest of the body is covered with small cycloid scales with reticulated striae. Lateral line originates approximately above pectoral fin and forms a straight line to caudal peduncle. There are 64*–*86 (76) scales along lateral line, 16*–*23 (20) along caudal peduncle and 16–20 (20) around caudal peduncle. Urogenital opening situated adjacent to origin of anal fin.

Rounded pectoral fin with 10 rays, extends just beyond origin of pelvic fin. Short pelvic fin with 6 rays. The first two dorsal and anal‐fin rays are unbranched, unsegmented and small. These two unbranched and unsegmented rays are followed by a single long unbranched and segmented ray, followed by numerous branched, segmented rays. The last dorsal‐ and anal‐fin ray is usually branched all the way to its base. Anal and dorsal fins set towards the posterior of body. Anal‐fin origin is 4–5 (5) fin rays anterior to the dorsal‐fin origin. Dorsal‐fin rays: 18*–*20 (18); anal‐fin rays: 22*–*24 (22). In both dorsal and anal fins, the anterior fin rays increase in length up to the fourth and fifth rays, then subsequent rays get progressively shorter. Caudal fin deeply forked with rounded lobes with bases covered in scales; distance from caudal‐fin flexion point to caudal‐fin tips is roughly equal to caudal peduncle length.

Live colour: body colour ranges from light to dark brown. Dorsal surface usually darker than the ventral surface. Thin curved vertical bars more conspicuous in the anterior portion of flank. Dark vertical bar originates from origin of dorsal fin, barely visible in darker specimens. A barely visible dark blotch is present near flexion point of the caudal fin. Fins brown. Preserved in ethanol: body colour ranges from light to dark brown. Dorsal surface usually darker than ventral surface. Dark vertical bar originates from origin of dorsal fin, barely visible in darker specimens. A barely visible dark blotch is present near flexion point of the caudal fin. Thin curved vertical bars conspicuous in the anterior portion of flank. Fins brown.

Total vertebrae: 41*–*42 (41), pre‐caudal vertebrae: 18*–*19 (19), caudal vertebrae: 22*–*24 (22), vertebrae at first dorsal radial: 20*–*21 (20), vertebrae at first anal radial: 19*–*20 (19).

Determining the sex of adults can be done externally by examining their anal‐fin base. In males, the body wall is dorsally indented, giving the dorsal margin of the anal fin a sigmoid curvature. In females, the body wall appears almost straight.

#### 4.5.6 Distribution

This species is found throughout the Okavango River, Angola and Namibia; see Figure 9.

#### 4.5.7 Etymology

The specific epithet is a noun in the genitive case of the Xanekwe people of Okavango River. The Xanekwe are one of the groups of people that have historically lived along the Okavango River and its delta. They traditionally were hunter gatherers and fishermen with close ties to the riverine environment.

#### 4.5.8 Remarks

*H. xanekweorum* and *H. tangwenai* have very similar morphology that makes these species difficult to distinguish.

### 4.6 *H. chilembwei* sp. nov

**FIGURE 14:**
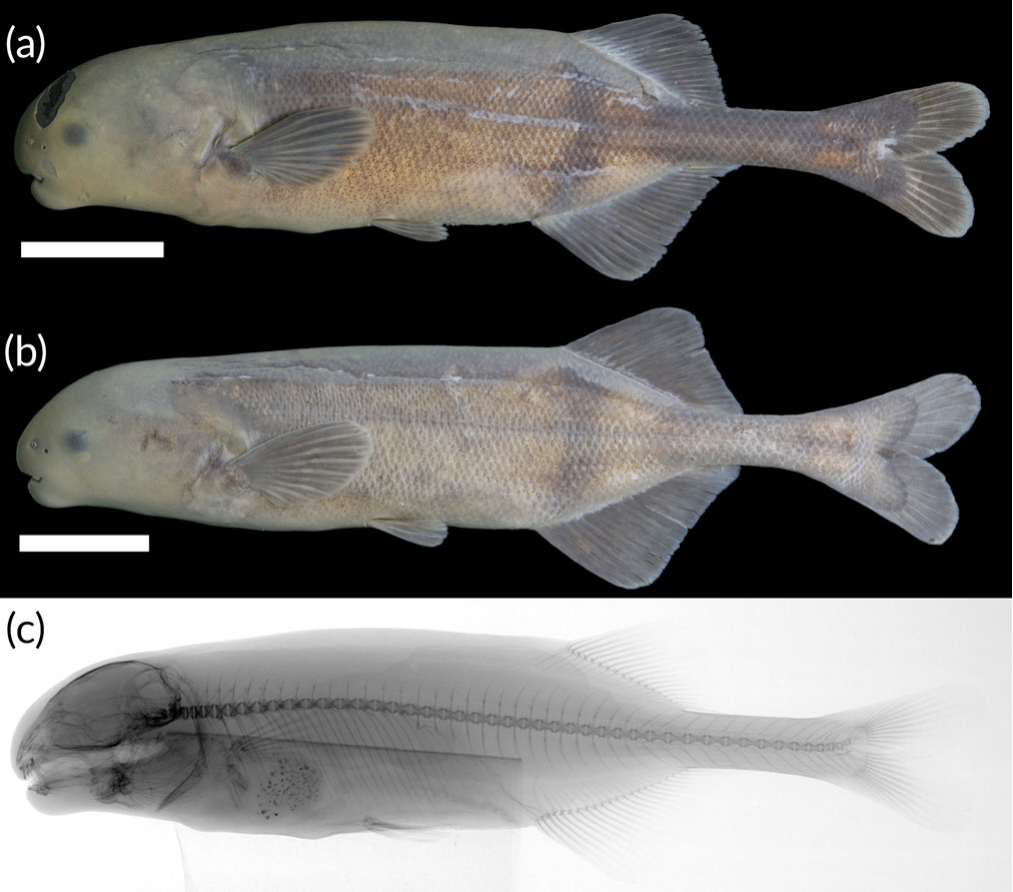
(a) Holotype of *Heteromormyrus chilembwei* sp. nov. (SAIAB 119004), 65.0 mm standard length (SL), from the Ruo River, lower Zambezi River system, Malawi, southern Africa; (b) paratype of *H. chilembwei* sp. nov. (SAIAB 119004), 69.0 mm SL, from the Ruo River, lower Zambezi River system, Malawi, southern Africa; (c) X‐ray radiograph of holotype. Scale bar =1 cm.

https://zoobank.org/NomenclaturalActs/2ba0ea02-8cdb-430b-99cb-0f2dac1a5cca; Figure 14.

*Hippopotamyrus* sp. ‘Ruo’ in Chakona et al. (2018).

*Hippopotamyrus* sp. ‘Ruo’ in Mutizwa et al. (2021).

*Heteromormyrus* sp. ‘Ruo’ in Sullivan et al. (2022).

#### 4.6.1 Holotype

SAIAB 246299, 65.1 mm SL, Lujeri tea estate, 16° 02.19′ S, 35° 39.80′ E, Ruo River, lower Zambezi River system, Malawi, D. Tweddle and T. Mäkinen, 21 May 2011, BOLD genseq‐1 COI: MAFW025‐11, GenBank genseq‐1 cyt *b*: MW600888, S7 intron: MW756346.

#### 4.6.2 Paratypes

SAIAB 119004, 3 specimens 48.0*–*69.0 mm SL, Lujeri tea estate, 16° 02.19′ S, 35° 39.80′ E, Ruo River, lower Zambezi River system, Malawi, D. Tweddle and T. Mäkinen, 21 May 2011, GenBank genseq‐2 S7 intron: MW756347; SAIAB 119013, 3 specimens 33.9*–*47.9 mm SL, Lujeri tea estate, Muluzi stream, lower Zambezi River system, Malawi, 16° 03.26′ S, 35° 40.27′ E, D. Tweddle and T. Mäkinen, 21 May 2011, BOLD genseq‐2 COI: MAFW039‐11, GenBank genseq‐2 cyt *b*: MW600889, S7 intron: MW756332, MW756333.

#### 4.6.3 Diagnosis

*H. chilembwei* sp. nov. possesses 16 scales around its caudal peduncle that further distinguish it from *H. tangwenai* sp. nov. (18*–*20), *H. tavernei* (20–22) and *H. longilateralis* (20). A high number of scales along the length of the caudal peduncle differentiate *H. chilembwei* sp. nov. (21*–*23) from *H. ansorgii* (16*–*19), *H. pauciradiatus* (14*–*18), *H. dolichorhynchus* (17–20) and *H. ndauorum* sp. nov. (16*–*20). A low number of anal‐fin rays separate *H. chilembwei* sp. nov. (23*–*25) from *H. angusticaudata* (28*–*30), *H. dolichorhynchus* (26*–*29) and *H. pappenheimi* (26*–*28). A low number of caudal vertebrae differentiated *H. chilembwei* sp. nov. (24–25) from *H. angusticaudata* (26*–*27). The high number of vertebrae before the first dorsal‐fin radial further distinguishes *H. chilembwei* sp. nov. (21*–*22) from *H. pappenheimi* (19). A higher number of vertebrae before the first anal‐fin radial separate *H. chilembwei* sp. nov. (20) from *H. pauciradiatus* (18*–*19) and *H. pappenheimi* (18). The number of anal‐fin rays between the leading ray and the one directly below the leading dorsal‐fin ray distinguishes *H. chilembwei* sp. nov. (6–7) from *H. xanekweorum* (4–5), *H. longilateralis* (3–5), *H. szaboi* (3–4) and *H. ndauorum* sp. nov. (3–4) with more anteriorly set dorsal fins. A narrow caudal peduncle distinguishes *H. chilembwei* sp. nov. (5.9%*–*6.2%SL) from *H. pauciradiatus* (7.3%–9.2%SL), *H. tavernei* (6.9%–8.6%SL), *H. longilateralis* (7.0%–8.2%SL), *H. szaboi* (6.4%–10.4%SL), *H. tangwenai* sp. nov. (6.3%–7.9%SL) and *H. ndauorum* sp. nov. (6.7%–7.5%SL). The snout length distinguishes *H. chilembwei* sp. nov. (42.6%–50.2%HL) from *H. ansorgii* (34.6%–41.4%HL), *H. pauciradiatus* (29.7%–41.0%HL), *H. tavernei* (24.7%–30.0%HL), *H. longilateralis* (38.3%–42.5%HL) and *H. angusticaudata* (39.5%–42.3%HL). The interorbital width distinguishes *H. chilembwei* sp. nov. (46.4%–51.6%HL) from *H. ansorgii* (28.6%–45.4%HL), *H. pauciradiatus* (29.7%–41.0%HL), *H. tavernei* (24.7%–30.0%HL), *H. longilateralis* (35.5%–43.7%HL), *H. dolichorhynchus* (34.8%–43.4%HL) and *H. angusticaudata* (38.1%–43.8%HL). *H. chilembwei* sp. nov. is readily distinguished from its congeners by its deep, rounded dorsal head profile and dark pectoral fins. The lack of clearly visible marks in the caudal peduncle further distinguishes *H. chilembwei* from *H. ansorgii*, *H. pauciradiatus* and *H. angusticaudata* that have a clearly visible dark blotch present near the flexion point of the caudal fin and a dark vertical bar in the caudal peduncle; *H. longilateralis*, *H. szaboi*, *H. tangwenai* and *H. xanekweorum* have only a dark blotch present near the flexion point of the caudal fin.

#### 4.6.4 Description

Proportional measurements and meristics are summarised in Table 5. Holotype meristic counts are presented in parentheses.

Rounded blunt snout, below eye level. Deep, rounded dorsal head profile that gradually becomes gently inclined like the rest of dorsal profile of the body. Small sub‐terminal mouth occurring below the level of pectoral‐fin base. Small rounded chin swelling transitions into concave ventral profile of the head. Round orbit. Anterior and posterior nostrils laterally positioned, closer to snout tip than opercular opening, anterior to and arranged horizontally in line with orbit. Anterior nostril positioned slightly higher than posterior nostril. Small gill opening with soft skin cover adjacent to pectoral‐fin base. Bicuspid teeth: 7*–*8 (7) in upper jaw and 8*–*9 (9) in lower jaw.

Laterally compressed body with greatest breadth occurring across gill covers. Fusiform body with greatest depth occurring roughly midway between the pectoral‐fin and pelvic‐fin origins. Body tapers gently towards head, while tapering sharply from origins of both dorsal and anal fins to produce a relatively thin caudal peduncle. Caudal peduncle maintains the same depth from its origin to roughly around half its length, then it gradually widens into two symmetrical lobes. Body covered with transparent membrane that becomes increasingly translucent to opaque towards head, dorsal and ventral surfaces in preserved specimens. Head without scales; rest of the body is covered in small cycloid scales with reticulated striae. Lateral line originates approximately above pectoral fin and forms a straight line to caudal peduncle. There are 82*–*84 (82) scales along the lateral line, 21*–*23 (22) along caudal peduncle and 16 around caudal peduncle. Urogenital opening situated adjacent to the origin of the anal fin.

Rounded pectoral fin with 10 rays, extends just beyond the origin of the pelvic fin. Short pelvic fin with 6 rays. The first two dorsal and anal‐fin rays are unbranched, unsegmented and small. These two unbranched and unsegmented rays are followed by a single long unbranched and segmented ray, followed by numerous branched, segmented rays. The last dorsal‐ and anal‐fin ray is usually branched all the way to its base. Anal and dorsal fins set towards the posterior of the body. Anal‐fin origin is 6–7 (7) fin rays anterior to the dorsal‐fin origin. Dorsal‐fin rays: 18*–*20 (20); anal‐fin rays: 23*–*25 (25). In both dorsal and anal fins, anterior fin rays increase in length up to the 4th and 5th rays then subsequent rays get progressively shorter. Caudal fin deeply forked with rounded lobes that have scaled bases; distance from caudal‐fin flexion point to caudal‐fin tips roughly equal to caudal peduncle length.

Body colour ranges from light to dark brown in specimens preserved in ethanol. Dorsal surface usually darker than ventral surface. Dark vertical bar originates from origin of dorsal fin, and it is barely visible in darker specimens. A barely visible dark blotch is present near flexion point of the caudal fin. Dark brown pectoral and pelvic fins. Dark caudal peduncle without any additional markings.

Total vertebrae: 43 (43), pre‐caudal vertebrae: 18–19 (18), caudal vertebrae: 24–25 (25), vertebrae at first dorsal radial: 21*–*22 (22), vertebrae at first anal radial: 20.

All *H. chilembwei* specimens examined in this study had a straight body wall at their anal‐fin base. Because no verification of the specimen's sex was carried out (e.g., by dissection of gonads), it is not possible to confirm whether these species were not sexually dimorphic. It is also possible that there were no sexually mature males among the examined specimens.

#### 4.6.5 Distribution

This species was collected from the Ruo River, a tributary of the Shire River, which flows into the lower Zambezi River system. The Ruo is a small montane river that flows off the Mulanje Massif, which is situated in the southeast corner of Malawi close to the Mozambique border (see Figure 9).

#### 4.6.6 Etymology

The specific epithet is a noun in the genitive case that honours the Reverend John Chilembwe, who led the first nationalist uprising in Malawi from 1914 to 1915 protesting against forced labour and African involvement in World War I.

### 4.7 *H. tangwenai* sp. nov

**FIGURE 15:**
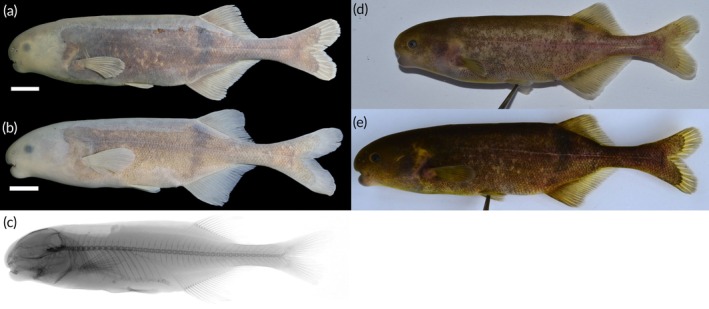
(a) Holotype of *Heteromormyrus tangwenai* sp. nov. male (SAIAB 200926), 102.2 mm standard length (SL), from the Nyamukombe River, Pungwe River system, Zimbabwe; (b) paratype of *H. tangwenai* sp. nov. female (SAIAB 200926), 69.0 mm SL, from the Nyamukombe River, Pungwe River system, Zimbabwe; (c) X‐ray radiograph of holotype; (d, e) live pictures of *H. tangwenai* sp. nov. Scale bar =1 cm.

https://zoobank.org/NomenclaturalActs/21a96ccb-2ff7-476b-996b-d9d6859819b4; Figure 15.

*Hippopotamyrus* sp. ‘Pungwe’ in Chakona et al. (2018).

*Hippopotamyrus* sp. ‘Pungwe’ in Mutizwa et al. (2021).

*Heteromormyrus* sp. ‘Pungwe’ in Sullivan et al. (2022).

#### 4.7.1 Holotype

SAIAB 246298, 102.2 mm SL, Nyamukombe River bridge in Sagambe, 18° 23′ 45.0″ S, 32° 58′ 14.6″ E, Pungwe River system, Zimbabwe, A. Chakona, W. Kadye and T. Bere, 11 December 2014, GenBank genseq‐1 COI: MH432084, cyt *b*: MW600887, S7 intron: MW756348.

#### 4.7.2 Paratypes

SAIAB 200926, 3 specimens 60.7–80.1 mm SL, Nyamukombe River bridge in Sagambe,18° 23′ 45.0″ S, 32° 58′ 14.6″ E, Pungwe River system, Zimbabwe, A. Chakona, W. Kadye and T. Bere, 11 December 2014, GenBank genseq‐2 COI: MH432085, S7 intron: MW756349.

#### 4.7.3 Non‐type specimens

SAIAB 201082, 2 specimens 26.2–69.0 mm SL, Nyamukwarara River next to the Nyamukwarara Clinic 18° 41′ 30.5″ S, 32° 55′ 25.0″ E, Pungwe River system, Zimbabwe, A. Chakona, W. Kadye and T. Bere, 17 December 2014, GenBank genseq‐3 COI: MH432078, MH432081; SAIAB 201091, 6 specimens 49.2–88.3 mm SL, Chiyengwa River just before confluence with Nyamukwarara River, 18° 41′ 16.0″ S, 32° 55′ 19.2″ E, Pungwe River system, Zimbabwe, A. Chakona, W. Kadye and T. Bere, 17 December 2014, GenBank genseq‐3 COI: MH432086, MH432083; SAIAB 201071, 5 specimens 22.8*–*66.5 mm SL, Makanga bridge on the road to Hauna, 18° 32′ 37.8″ S, 32° 48′ 04.6″ E, Pungwe River system, Zimbabwe, A. Chakona, W. Kadye and T. Bere, 16 December 2014, GenBank genseq‐3 COI: MH432079; SAIAB 201030, 5 specimens, 24.8–83.9 mm SL, Rwera River through Marupeti shops at Zimbabwe and Mozambique border, 18° 32′ 36.4″ S, 32° 48′ 5.8″ E, Pungwe River system, Zimbabwe, A. Chakona, W. Kadye and T. Bere, 14 December 2014, GenBank genseq‐3 COI: MH432077, cyt *b*: MW600885, S7 intron: MW756328, MW756329; SAIAB 201074, 1 specimen 78.1 mm SL, Pungwe River mainstem by the abandoned church, 18° 26′ 29.1″ S, 32° 53′ 14.9″ E, Pungwe River system, Zimbabwe, A. Chakona, W. Kadye and T. Bere, 16 December 2014, GenBank genseq‐3 COI: MH432080; SAIAB 201044, 1 specimen 86.9 mm SL, Nyamukombe River bridge between Chisuko School and Katiyo, 18° 22′ 55.7″ S, 33° 01′ 57.8″ E, Pungwe River system, Zimbabwe, A. Chakona, W. Kadye and T. Bere, 14 December 2014, GenBank genseq‐3 COI: MH432076, cyt *b*: MW600884, S7 intron: MW756350, MW756351; SAIAB 205084, 1 specimen 62.0 mm SL, from bridge on road to Gatsi, below falls, 18° 31.945′ S, 032° 48.452′ E, Pungwe River system, Zimbabwe, A. Chakona, T. Bere and M. Machingura, 13 December 2013, GenBank genseq‐3 COI: MH432071; SAIAB 201021, 4 specimens 55.9–104.9 mm SL, bridge on road to Honde mission, 18° 32′ 37.5″ S, 32° 48′ 15.8″ E, Pungwe River system, Zimbabwe, A. Chakona, W. Kadye, T. Bere, M. Machingura, 13 December 2013, GenBank genseq‐3 COI: MH432082.

#### 4.7.4 Diagnosis

*H. tangwenai* sp. nov. possesses a higher number of scales around its caudal peduncle (18*–*20), differentiating it from *H. ndauorum* sp. nov. (16), *H. chilembwei* (16), *H. ansorgii* (14*–*16), *H. dolichorhynchus* (12*–*16) and *H. pappenheimi* (12*–*14). A lower number of anal‐fin rays further separate *H. tangwenai* sp. nov. (20*–*25) from *H. angusticaudata* (28*–*30) and *H. dolichorhynchus* (26*–*29). The number of anal‐fin rays between the leading ray and the one directly below the leading dorsal‐fin ray in *H. tangwenai* sp. nov. (6–7) distinguishes it from *H. xanekweorum* (4–5), *H. longilateralis* (3–5), *H. szaboi* (3–4) and *H. ndauorum* sp. nov. (3–4). *H. tangwenai* sp. nov. (21.0%*–*23.9%SL) has a narrower body depth compared to *H. pauciradiatus* (23.9%*–*27.9%SL). *H. tangwenai* sp. nov. is distinguished from *H. tavernei* by interorbital width (41.7%–54.4% vs. 24.7%–30.0%HL), snout length (41.1%–53.5% vs. 24.7%–30.0%HL) and head length (21.6%–24.9% vs. 25.8%–29.2%SL). *H. tangwenai* sp. nov. has fewer vertebrae (41*–*42) compared to *H. chilembwei* (43) and *H. angusticaudata* (46*–*47). *H. tangwenai* sp. nov. is further differentiated from *H. angusticaudata* by a low number of caudal vertebrae (22*–*25 vs. 26*–*27), fewer vertebrae before the first dorsal radial (19*–*21 vs. 22*–*24) and fewer vertebrae before the first anal‐fin radial (18–20 vs. 21*–*22). *H. tangwenai* sp. nov. has a rounded head profile that distinguishes it from *H. xanekweorum*, *H. szaboi* and *H. longilateralis* that have a flat head profile that forms an obtuse angle with the rest of the body profile. *H. tangwenai* sp. nov. has a dark blotch near flexion point of the caudal fin which distinguishes it from *H. dolichorhynchus*, *H. chilembwei* sp. nov. and *H. ndauorum* sp. nov. that do not have any clearly visible marks in the caudal peduncle; *H. ansorgii*, *H. pauciradiatus* and *H. angusticaudata* have a clearly visible dark blotch present near the flexion point of the caudal fin and a dark vertical bar in the caudal peduncle.

#### 4.7.5 Description

Proportional measurements and meristics are summarised in Table 5. Holotype meristic counts are presented in parentheses.

Rounded blunt snout, below eye level. Deep, rounded dorsal head profile that gradually becomes gently inclined like the rest of the dorsal profile of the body. Small sub‐terminal mouth occurring below the level of pectoral‐fin base. Small rounded chin swelling transitions into concave ventral profile of head. Round orbit. Anterior and posterior nostrils laterally positioned, closer to snout tip than opercular opening, anterior to and arranged horizontally in line with orbit. Anterior nostril positioned slightly higher than posterior nostril. Small gill opening with soft skin cover adjacent to pectoral fin‐base. Bicuspid teeth: 7*–*8 (7) in upper jaw and 8*–*9 (8) in lower jaw.

Laterally compressed body with greatest breadth occurring across gill covers. Fusiform body with greatest depth occurring between the origin of dorsal and anal fins. Body tapers gently towards head, while tapering sharply from origins of both dorsal and anal fins to produce a relatively thin caudal peduncle. Caudal peduncle maintains the same thin depth from its origin to roughly around half its length, then it gradually widens into two symmetrical lobes. Body covered with transparent membrane that becomes increasingly translucent to opaque towards head, dorsal and ventral surfaces in preserved specimens. Head without scales; rest of the body is covered with small cycloid scales with reticulated striae. Lateral line originates approximately above pectoral fin and forms a straight line to the caudal peduncle. There are 71*–*83 (83) scales along the lateral line, 18–22 (21) along caudal peduncle and 18–20 (20) around caudal peduncle. Urogenital opening situated adjacent to origin of anal fin.

Rounded pectoral fin with 10 rays, extends just beyond the origin of the pelvic fin. Short pelvic fin with 6 rays. The first two dorsal‐ and anal‐fin rays are unbranched, unsegmented and small. These two unbranched and unsegmented rays are followed by a single long unbranched and segmented ray, followed by numerous branched, segmented rays. The last dorsal‐ and anal‐fin ray is usually branched all the way to its base. Anal and dorsal fins set towards the posterior of the body. Anal‐fin origin is 6–7 (6) fin rays anterior to the dorsal‐fin origin. Dorsal‐fin rays: 17*–*20 (18); anal‐fin rays: 20*–*25 (22). In both dorsal and anal fins, the anterior fin rays increase in length up to the fourth and fifth rays, then subsequent rays get progressively shorter. Caudal fin deeply forked with rounded lobes with bases covered in scales; distance from caudal‐fin flexion point to caudal‐fin tips is roughly equal to caudal peduncle length.

Live colour: body colour ranges from light to dark brown. Body surface with irregular patches of light and dark scales giving a mottled pattern in some specimens, whereas others lack this pattern. Dorsal surface usually darker than the ventral surface. Dark vertical bar originates from origin of dorsal fin, barely visible in darker specimens. Dark vertical bar originates from origin of dorsal fin, barely visible in darker specimens. Barely visible dark blotch is present near flexion point of the caudal fin. Fins brown usually a lighter shade than the body. Preserved in ethanol: body colour ranges from light to dark brown. Body surface with irregular patches of light and dark scales giving a mottled pattern. Dorsal surface usually darker than ventral surface. Dark vertical bar originates from origin of dorsal fin, barely visible in darker specimens. Barely visible dark blotch is present near flexion point of the caudal fin. Fins brown, usually a lighter shade than the body.

Total vertebrae: 41*–*42 (41), pre‐caudal vertebrae: 17–19 (19), caudal vertebrae: 22*–*25 (24), vertebrae at first dorsal radial: 19*–*21 (20), vertebrae at first anal radial: 18*–*20 (18).

Determining the sex of adults can be done externally by examining their anal‐fin base. In males, the body wall is dorsally indented, giving the dorsal margin of the anal fin a sigmoid curvature. In females, the body wall appears almost straight.

#### 4.7.6 Distribution

This species was collected in the headwater streams of the Pungwe River, Zimbabwe; see Figure 9.

#### 4.7.7 Etymology

The specific epithet is a noun in the genitive case honouring Chief Rekayi Tangwena (1910–June 1984), a traditional chief of the Tangwena people of Nyanga in the Manicaland Province of Zimbabwe. Chief Tangwena fiercely resisted the unlawful eviction of his people from their ancestral lands by the white minority settler government. He remains a celebrated national hero for his efforts in the liberation of Zimbabwe.

### 4.8 *H. ndauorum* sp. nov

**FIGURE 16:**
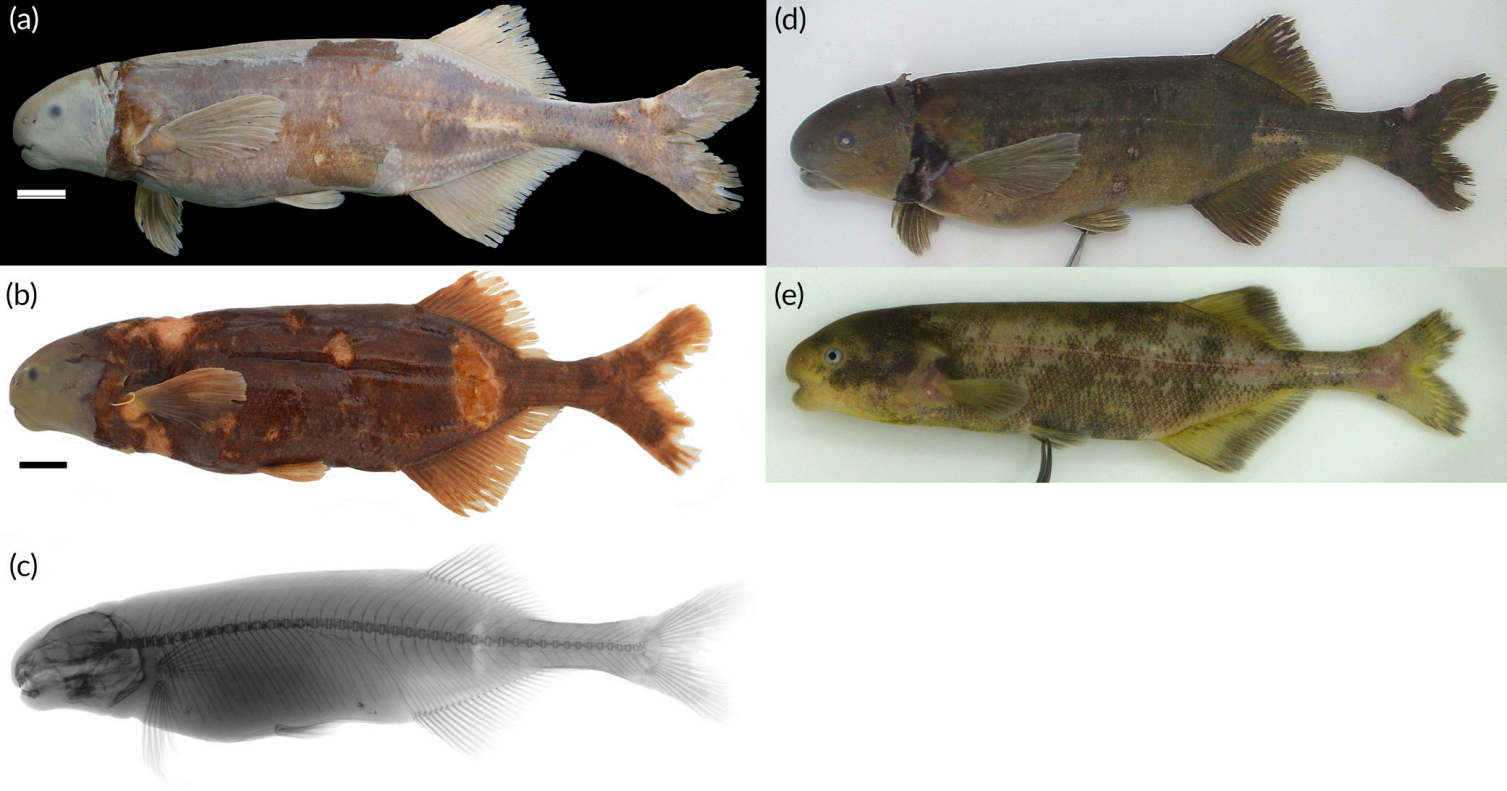
(a) Holotype of *Heteromormyrus ndauorum* sp. nov. (SAIAB 67420), 149.0 mm standard length (SL), from the Mudzira River, Buzi River system, Zimbabwe and Mozambique; (b) paratype of *H. ndauorum* sp. nov. (SAIAB 67420), 135.0 mm SL, from the Mudzira River, Buzi River system, Zimbabwe and Mozambique; (c) X‐ray radiograph of holotype; (d, e) live pictures of *H. ndauorum* sp. nov. Scale bar =1 cm.

https://zoobank.org/NomenclaturalActs/af945b25-a685-4089-b168-711b77e7c7c4; Figure 16.

*Hippopotamyrus* sp. ‘Buzi’ in Chakona et al. (2018).

*Hippopotamyrus* sp. ‘Buzi’ in Mutizwa et al. (2021).

*Heteromormyrus* sp. ‘Buzi’ in Sullivan et al. (2022).

#### 4.8.1 Holotype

SAIAB 246297, 149.0 mm SL, Mudzira River below Muhate, 19.6750° S, 33.1910° E, Buzi River system, Mozambique, R. Bills, S. Chimela, A. Chivindzi, 20 September 2002, BOLD genseq‐1 COI: SAFW886‐14.

#### 4.8.2 Paratypes

SAIAB 67420, 2 specimens 135.0–149.0 mm SL, Mudzira River below Muhate, 19.6750° S, 33.1910° E, Buzi River system, Mozambique, R. Bills, S. Chimela and A. Chivindzi on 20 September 2002; SAIAB 205113, 4 specimens 30.0–62.0 mm SL, Rusitu River bridge, 20° 03.519′ S, 32° 51.315′ E, Buzi River system, Zimbabwe, A. Chakona, W. Kadye, T. Bere, M. Machingura,17 December 2013, GenBank genseq‐2 COI: MH432072, MH432074; cyt *b*: MW600901, MW600902, S7 intron: MW756340, MW756341, MW756338, MW756339.

#### 4.8.3 Diagnosis

The number of anal‐fin rays between the leading ray and the one directly below the leading dorsal‐fin ray in *H. ndauorum* sp. nov. (3–4) distinguishes it from *H. pappenheimi* (6–9), *H. pauciradiatus* (6–9), *H. ansorgii* (6–9), *H. dolichorhynchus* (5–7), *H. angusticaudata* (5–6), *H. tangwenai* sp. nov. (6–7) and *H. chilembwei* sp. nov. (6–7). *H. ndauorum* sp. nov. possesses 16 scales around its caudal peduncle that distinguish it from *H. tangwenai* (18*–*20), *H. tavernei* (20–22) and *H. longilateralis* (20). A lower number of anal‐fin rays separate *H. ndauorum* sp. nov. (23–24) from *H. angusticaudata* (28*–*30), *H. dolichorhynchus* (26*–*29) and *H. pappenheimi* (26*–*28). A higher number of vertebrae differentiate *H. ndauorum* sp. nov. (42) from *H. szaboi* (40–41), *H*. *chilembwei* (43) and *H. angusticaudata* (46*–*47). *H. ndauorum* sp. nov. is further separated from *H. chilembwei* by having fewer scales along its lateral line (72*–*78 vs. 82*–*84), as well as fewer scales along the length of the caudal peduncle (16*–*20 vs. 21*–*23). Lack of clearly visible marks on caudal peduncle distinguishes *H. ndauorum* sp. nov. from *H. ansorgii*, *H. pauciradiatus* and *H. angusticaudata* that have a clearly visible dark blotch near the flexion point of the caudal fin and a dark vertical bar in the caudal peduncle; *H. longilateralis*, *H. szaboi*, *H. tangwenai* and *H. xanekweorum* have a dark blotch present near the flexion point of the caudal fin.

#### 4.8.4 Description

Proportional measurements and meristics are summarised in Table 5. Holotype meristic counts are presented in parentheses.

Rounded blunt snout, below eye level. Straight dorsal head profile forms obtuse angle with the gently inclined dorsal body profile. Small sub‐terminal mouth occurring below the level of the pectoral‐fin base. Small rounded chin swelling transitions into concave ventral profile of head. Round orbit. Anterior and posterior nostrils laterally positioned, closer to snout tip than opercular opening, anterior to and arranged horizontally in line with orbit. Anterior nostril positioned slightly higher than posterior nostril. Small gill opening with soft skin cover adjacent to pectoral‐fin base. Bicuspid teeth: 7*–*9 (7) in upper jaw and 8*–*9 (8) in lower jaw.

Laterally compressed body with greatest breadth occurring across gill covers. Fusiform body with greatest depth occurring between origin of dorsal and anal fins. Body tapers gently towards head while tapering sharply from origins of both dorsal and anal fins to produce a relatively thin caudal peduncle. Caudal peduncle maintains the same thin depth from its origin to roughly around half its length, then it gradually widens into two symmetrical lobes. Body covered with transparent membrane that becomes increasingly translucent to opaque towards head, dorsal and ventral surfaces in preserved specimens. Head without scales; rest of the body is covered with small cycloid scales with reticulated striae. Lateral line originates approximately above pectoral fin and forms a straight line to the caudal peduncle. There are 72*–*78 (78) scales along the lateral line, 16*–*20 (16) along caudal peduncle length and 16 around caudal peduncle. Urogenital opening is situated adjacent to the origin of anal fin.

Rounded pectoral fin with 10 rays, extends just beyond the origin of the pelvic fin. Short pelvic fin with 6 rays. The first two dorsal‐ and anal‐fin rays are unbranched, unsegmented and small. These two unbranched and unsegmented rays are followed by a single long unbranched and segmented ray, followed by numerous branched, segmented rays. The last dorsal‐ and anal‐fin ray is usually branched all the way to its base. Anal and dorsal fins set towards the posterior of the body. Anal‐fin origin is 3–4 (4) fin rays anterior to the dorsal‐fin origin. Dorsal‐fin rays: 19*–*20 (20); anal‐fin rays: 23*–*24 (23). In both dorsal and anal fins, the anterior fin rays increase in length up to fourth and fifth rays, then subsequent rays get progressively shorter. Caudal fin deeply forked with rounded lobes that have scaled bases; distance from caudal‐fin flexion point to caudal‐fin tips is roughly equal to caudal peduncle length.

Live colour: body colour ranges from light to dark brown. Body surface with irregular patches of light and dark scales giving a mottled pattern in some specimens, whereas others lack this pattern. Dorsal surface usually darker than the ventral surface. Dark vertical bar originates from the origin of dorsal fin, barely visible in darker specimens. Dark vertical bar originates from the origin of dorsal fin, barely visible in darker specimens. Barely visible dark blotch is present near flexion point of the caudal fin. Fins brown, usually a lighter shade than the body. Preserved in ethanol: body colour dark brown. Dorsal surface usually darker than ventral surface. Dark vertical bar originates from the origin of dorsal fin, and it is barely visible in darker specimens. Barely any visible markings in the caudal peduncle.

Total vertebrae: 41–43 (42), pre‐caudal vertebrae: 17–18 (18), caudal vertebrae: 24–25 (24), vertebrae at first dorsal radial: 19–20 (20), vertebrae at first anal radial: 18–19 (18).

All the specimens of *H. ndauorum* examined in this study had a straight body wall at their anal‐fin base. Because no verification of the sex of the specimens was carried out (e.g., by dissection of gonads), it is not possible to confirm whether this species is not sexually dimorphic. It is also possible that there was no sexually mature males among the examined specimens.

#### 4.8.5 Distribution

This species was collected in the head water streams of the Buzi River, Mozambique and Zimbabwe; see Figure 9.

#### 4.8.6 Etymology

The specific epithet is a noun in the genitive case that honours the Ndau people, who have historically lived in the Chimanimani district of Zimbabwe, where the species was collected.

### 4.9 Identification key for southern African *Heteromormyrus* species based on external characters

1a. Nostrils aligned horizontally below the level of the orbit            *H. dolichorhynchus* sp. nov. (Figure 6a).

1b. Anterior nostril was always positioned higher than the posterior nostril and both nostrils horizontally in line with the orbit (Figure 6b,c)                        2

2a. Clearly visible dark blotch is present near the flexion point of the caudal fin (Figure 7b,c)                  3

2b. Barely visible blotch in the caudal peduncle (Figure 7a)                         9

3a. Presence of dark vertical bar just anterior to the blotch near the flexion point of the caudal fin (Figure 7c)          4

3b. Absence of a dark vertical bar just anterior to the blotch near the flexion point of the caudal fin               6

4a. Presence of a series of thin slightly curved vertical bars more conspicuous in the anterior portion of flank (Figure 7d)              *H. angusticaudata* sp. nov.

4b. Absence of a series of thin slightly curved vertical bars (Figure 7b,c)                        5

5a. Deep caudal peduncle (7.3%–9.2%SL)    *H. pauciradiatus*

5b. Slender caudal peduncle (6.0%–7.2%SL)    *H. ansorgii*

6a. Number of anal‐fin rays between the leading ray and the one directly below the leading dorsal‐fin ray 6–7   *H. tangwenai* sp. nov.

6b. Number of anal‐fin rays between the leading ray and the one directly below the leading dorsal‐fin ray 3–5           7

7a. Head profile rounded (Figure 6c)         *H. szaboi*

7b. Head profile forming an obtuse angle with the rest of the dorsal body surface (Figure 6a)                   8

8a. Slender caudal peduncle (5.0%–6.7%SL)   *H. xanekweorum* sp. nov.

8b. Deep caudal peduncle (7.2%–8.2%SL)     *H. longilateralis*

9a. 12–14 scales around the caudal peduncle  *H. pappenheimi*

9b 16–18 scales around the caudal peduncle        10

10a. Slender caudal peduncle (5.9%–6.2%SL)   *H. chilembwei* sp. nov.

10b. Deep caudal peduncle (6.9%–7.5%SL)   *H. ndauorum* sp. nov.

## 5 DISCUSSION

### 5.1 Assignment of lineages to available names

Prior to this study, the genus *Heteromormyrus* was represented by three species in the Kwanza River system: *H. ansorgii*, *H. pauciradiatus* and *H. pappenheimi*. Sullivan et al. (2022) provided molecular evidence, showing that *Heteromormyrus* sp. ‘K4’, identified by Mutizwa et al. (2021), is conspecific with *H. pauciradiatus*. However, there were no genetic data available from the type material of *H. ansorgii* and *H. pappenheimi*. Therefore, the morphological analysis conducted in the present study was crucial for determining lineages that are likely to be conspecific with these two species. Specimens belonging to *Heteromormyrus* sp. ‘K3’ were morphologically comparable to the syntypes of *H. ansorgii*. We therefore concluded that this lineage likely represents *H. ansorgii* s.s, further supporting the previous determination by Mutizwa et al. (2021) that the Kwanza River system is the most likely type system for this species. We compared the morphological characteristics for the remaining lineages from the Kwanza River system with the original description and the syntypes of *H. pappenheimi* to determine if any of these lineages could be conspecific with this species. None of these lineages were morphologically comparable to *H. pappenheimi*, indicating that fresh material for this species was not included in the molecular study by Mutizwa et al. (2021). *H. pappenheimi* was originally described based on specimens that were collected at Cunga in the lower Kwanza River. Future surveys targeting the lower Kwanza River, a system that remains poorly sampled, are likely to reveal additional diversity within the genus *Heteromormyrus* and possibly find topotypes of *H. pappenheimi*. Future studies that will successfully extract and sequence DNA from the syntypes of *H. ansorgii* and *H. pappenheimi*, following the approach used by Sullivan et al. (2022), may offer a way to link these historical specimens to freshly collected specimens and provide insights on the evolutionary relationships of these species.

### 5.2 Mormyrid diversity in the Kwanza River basin

The ichthyofauna of the Kwanza River system remains poorly documented because exploration was curtailed by 27 years of civil unrest. However, this has changed over the past decade, where international support for environmental conservation and research has grown (Huntley & Ferrand, 2019). This growth is reflected by collaborative research efforts in the country soon after the end of the civil war and the subsequent signing of memoranda of understanding between the Angolan Ministry of Environment, the Instituto Superior de Ciências da Educação (ISCED) and the South African National Biodiversity Institute (SANBI), in 2009, which paved the way for further cooperative biodiversity projects, including training exercises and rapid biodiversity assessments (Huntley & Ferrand, 2019; Rejmánek et al., 2017). This has also facilitated important collaborative expeditions, such as Kwanza River project (2007–2009), supported by Instituto Nacional de Investigação Pesqueira (INIP) in Angola, the NRF‐SAIAB and, more recently, the National Geographic Okavango Wilderness Project (NGOWP 2018). A number of new species have been described based on these collections, with representatives in the Amphilidae, *Amphilius pagei* Thomson & Swartz, 2018, Cichlidae, *Serranochromis cuanza* Stauffer et al., 2021, *Serranochromis swartzi* Stauffer et al., 2021; Procatopodidae, ‘*Lacustricola*’ *pygmaeus* Bragança et al., 2021; and the Anabantidae, *Microctenopoma steveboyesi* Skelton et al., 2021. The present study has increased the number of mormyrid species in the Kwanza River from seven to nine. This number is likely to increase as more surveys are undertaken and other mormyrid groups examined, as evidenced by findings of more species in adjacent basins that have been relatively well studied compared to the Kwanza River system (e.g., Kramer et al., 2004, 2016; Kramer & Swartz, 2010; Kramer & van der Bank, 2011). This recent discovery of new fish species in the Kwanza River basin indicates that it could possibly be a previously overlooked biodiversity and endemic hotspot.

### 5.3 Diversity and distribution of *Heteromormyrus* in southern Africa

The species from the genus *Heteromormyrus* are found in the Congo, Kwanza, Kunene, Okavango, Zambezi, Pungwe and Buzi River basins. The distribution of these species across multiple rivers suggests historical riverine connections, with subsequent isolation resulting in allopatric speciation. The earliest common ancestor of the Mormyridae is estimated to have between 82.92 and 67.53 million years old (Ma) with the clade, including *Heteromormyrus* diverging around 15.1*–*11 Ma (Peterson et al., 2022). The clade that contained *Heteromormyrus* is thought to have rapidly diversified primarily in the Congo River basin (Peterson et al., 2022). The relatively close proximity of some of the southern tributaries of the Congo River to the northern tributaries of the Kwanza River, such as the Lucala River, as well as the shared fish fauna between these river systems, suggests an exchange of species between these rivers, probably by headwater capture (Skelton, 2019). The early exchange of the fish species between the Kwanza and Congo rivers, as well as subsequent speciation, may explain the high number of *Heteromormyrus* species in the Kwanza River, as well as the more basal lineages represented by some of these species (e.g., *Heteromormyrus* sp. ‘Inkisi River’, *H. dolichorhynchus* and *H. pauciradiatus*) (Mutizwa et al., 2021; Sullivan et al., 2022).

Some of the species in the Kwanza River system, for instance, *H dolichorhynchus*, *H. pauciradiatus* and *H. ansorgii* s.s. occur in sympatry. The EODs produced by mormyrids have been described as a key innovation that allows them to communicate with relatively fewer constraints from the environment, predators and competing signallers (Carlson & Arnegard, 2011). This results in communication with reduced distortion from the environment unlike other means of communication, for example, acoustic communication. Thus, mormyrids may be more sensitive to even small differences in pulse waveforms, for instance, between males and females or between sister species. This unique communication facilitates sexual selection and has been shown to evolve at a much faster rate compared to other traits, such as morphological and trophic ecological traits, making it an important early driver of species divergence even among sympatric species (Arnegard et al., 2005; Carlson & Arnegard, 2011; Feulner et al., 2007; Lavoué et al., 2008). The development of unique EODs may have driven the divergence of the multiple sympatric species within the Kwanza River. Although this study does not provide EOD evidence to test this hypothesis, evidence from *H. szaboi* in the upper Zambezi River shows that it consists of at least three morphologically indistinguishable lineages with relatively low genetic divergence, but with distinct EODs that are thought to be in the early stages of species divergence (Kramer et al., 2004; Sullivan et al., 2022). Further investigations into the factors shaping the diversity and distribution patterns of *Heteromormyrus* will need to integrate EODs, morphological and trophic ecological traits.

### 5.4 Conservation implications

Prior to this study, the taxonomy of the *Heteromormyrus* in southern Africa was poorly known, as there were only five valid species recognised in this region (*H. pauciradiatus*, *H. ansorgii*, *H. szaboi*, *H. longilateralis* and *H. pappenheimi*). *H. ansorgii* acted as catch‐all species name previously assigned to specimens of this genus collected from southern Africa. Consequently, this species was thought to have a distribution spreading across several southern African river systems, which resulted in it being classified as least concern on the International Union for Conservation of Nature (IUCN) Red list of threatened species (Bills et al., 2018). *H. pappenheimi* and *H. szaboi* are considered to be of least concern due to presumed wide distribution ranges in a habitat considered to be healthy, whereas *H. pauciradiatus* is classified as data deficient due to the species being known from a single site prior to this study (Da Costa, 2007; Tweddle, 2007). This illustrates the critical gap in knowledge of many of the freshwater fish species inhabiting this river system. This study described six new species that were previously included under a single species, *H. ansorgii*, indicating that the conservation statuses of these species will need to be determined.

Because species in the genus *Heteromormyrus* are associated with lotic habitats in perennial streams and rivers (Ford & Albert, 2022), they are likely to face similar threats associated with these habitats. In southern Africa, increased anthropogenic pressure from impoundments and water abstraction altering the natural river flow (e.g., Kwanza and Kunene River), alteration of the riparian zone and chemical inputs from the farming activities (e.g., Ruo River), illegal mining activities, deforestation and introduction of non‐native fish species (e.g., Kwanza, Pungwe and Buzi Rivers) have been identified as some of the major threats to the aquatic fauna (Chakona et al., 2018; de Moor et al., 2000; Kadye & Chakona, 2013; Skelton, 2019). The ecology of the species identified in this study remains as a gap that future studies need to address to provide an understanding of how these threats impact the species and to assist with formulating effective conservation recommendations.

## 6 CONCLUSION

This study provided formal descriptions for six new species from the genus *Heteromormyrus* based on the genetic evidence provided by Mutizwa et al. (2021) and the morphological data from the present study. Two of the new species *H. dolichorhynchus* and *H. angusticaudata* were described from the Kwanza River, whereas *H. xanekweorum*, *H. chilembwei*, *H. tangwenai* and *H. ndauorum* were each described from the Okavango, Ruo, Pungwe and Buzi rivers, respectively. The description of six new species from the *H. ansorgii* species complex shows that the previous wide distribution pattern attributed to this species was due to incomplete taxonomic knowledge. The formal recognition of these morphologically similar species is a fundamental first step for further research and conservation efforts involving these species. Additional taxonomic work within this genus remains essential, as the limited number of samples prevented further exploration into the taxonomic statuses of other undescribed species of the genus *Heteromormyrus* (e.g., *H*. sp. ‘K2’ from the Kwanza, *H*. sp. ‘UZ1’ and ‘HaZ’ from the Zambezi River, ‘HaK’ from the Kwando River and *H*. sp. ‘Insiki River’ from the Insiki River) identified in other studies of this group (Kramer et al., 2004; Mutizwa et al., 2021; Sullivan et al., 2022). *H. pappenheimi* remains a valid species from the Kwanza River; however, there is a need for collection of additional voucher and genetic material for this species to define its phylogenetic relationships with its congeners, as well as providing an updated species description. Despite the limitations in addressing some of the known lineages, this study increased the number of described mormyrid species within southern Africa, as well as reduced some of the ambiguity surrounding the overall diversity of freshwater fish diversity in this region.

## AUTHOR CONTRIBUTIONS

Project conceptualisation: Albert Chakona and Wilbert T. Kadye; data generation: Tadiwa I. Mutizwa and Pedro H. N. Bragança; data analysis: Tadiwa I. Mutizwa; manuscript preparation: Albert Chakona, Pedro H. N. Bragança, Tadiwa I. Mutizwa and Wilbert T. Kadye; funding: Wilbert T. Kadye and Albert Chakona.

## FUNDING INFORMATION

This research was supported by the Rhodes University Sandisa Imbewu Grant, the NRF‐Research Development Grant (CSRP190416431023), NRF‐SAIAB Refresh project (FBIP‐211006643719) and NRF‐SAIAB topotypes project (IBIP‐BS 13100251309).
